# Exploration of *Amomum cardamomum* Seed-Derived Compounds: LC-MS Profiling, Network Pharmacology, and Computational Analyses for Innovative Multi-Targeted Breast Cancer Therapy

**DOI:** 10.12688/f1000research.168214.1

**Published:** 2025-12-08

**Authors:** Dessy Arisanty, Salsabila Putri Khairani, Kevin Nathaniel Cuandra, Aldi Tamara Rahman, Christopher Daniel Tristan, Angela Stephanie Yahono, Muhammad Samudra Ilham, Ajib Zaim Alamsyah, Bezalel Tioleyson Sarjono, Daivan Febri Juan Setiya, Phelia Klarissa Huang, Alya Jihan Hafidza, Afiq Triyandi Hakam, Muhammad Farid Hamka, Daffa Zachary Rasendriya, Muhammad Naufal Hibatullah, Kuni Zakiyyah Sumargo

**Affiliations:** 1 Biochemistry, Universitas Andalas Faculty of Medicine, Padang, West Sumatra, Indonesia; 2 Biomedical Science, Universitas Andalas Faculty of Medicine, Padang, West Sumatra, Indonesia; 3 Medicine, Universitas Andalas Faculty of Medicine, Padang, West Sumatra, Indonesia; 4 Obstetrics and Gynecology, Cipto Mangunkusumo Hospital, Jakarta, Special Capital Region of Jakarta, Indonesia; 5 Medicine, Universitas Sebelas Maret Faculty of Medicine, Surakarta, Central Java, Indonesia; 6 Medicine, Universitas Gadjah Mada Faculty of Medicine Public Health and Nursing, Sleman, Special Region of Yogyakarta, Indonesia; 7 Biology, Universitas Andalas Faculty of Mathematics and Natural Sciences, Padang, West Sumatra, Indonesia; 8 Medicine, Universitas Udayana Faculty of Medicine, Denpasar, Bali, Indonesia; 9 Medicine, Universitas Sam Ratulangi Faculty of Medicine, Manado, North Sulawesi, Indonesia; 10 Medicine, Universitas Islam Indonesia Faculty of Medicine, Sleman, Special Region of Yogyakarta, Indonesia; 11 Nutritional Science, Universitas Pembangunan Nasional Veteran Jakarta Faculty of Health Science, Jakarta, Special Capital Region of Jakarta, Indonesia; 12 Medicine, Universitas Sumatera Utara Faculty of Medicine, Medan, North Sumatra, Indonesia; 13 Medicine, Universitas Jember Faculty of Medicine, Jember, East Java, Indonesia; 14 Medicine, Universitas Negeri Gorontalo Faculty of Medicine, Gorontalo City, Gorontalo, Indonesia

**Keywords:** Amomum cardamomum, breast cancer, LC-MS, network pharmacology, molecular docking, drug discovery

## Abstract

**Background:**

Breast cancer remains one of the leading causes of cancer-related mortality worldwide, and the emergence of drug resistance, systemic toxicity, and limited efficacy of current therapies highlight the need for safer and more effective treatment. Natural products have emerged as promising sources of multi-target anticancer agents. *A. cardamomum* has demonstrated preliminary anticancer potential, yet the bioactive constituents and their molecular mechanisms in breast cancer remain poorly elucidated.

**Methods:**

This study integrated *in silico* approaches to investigate the therapeutic potential of *A. cardamomum* seed extract against breast cancer. LC–MS analysis identified phytochemical compounds, followed by network pharmacology to determine their potential targets and molecular pathways. Pharmacokinetic and toxicity predictions were assessed through ADMET and Lipinski’s rule of five analyses to evaluate drug-likeness and safety. Molecular docking and molecular dynamics (MD) simulations were conducted to evaluate binding affinity and structural stability of compounds with key oncogenic proteins.

**Results:**

LC-MS profiling identified 22 distinct compounds in *A. cardamomum* seeds. ADMET and Lipinski analyses demonstrated that most compounds possessed high gastrointestinal absorption, favorable oral bioavailability, and low toxicity risk. Network pharmacology highlighting SRC, TNF-α, Caspase-3, and EGFR as central nodes in the protein-protein interaction network. Molecular docking identified compounds C17 and C20 as the most promising bioactives, showing strong binding affinities and interactions similar to control ligands. MD simulations confirmed their stable complexes, indicating conformational stability and robust ligand–protein interactions.

**Conclusion:**

This study highlights the promising multi-target anticancer potential of *A. cardamomum* seeds. Compounds C17 and C20 were identified as lead candidates with strong and stable interactions with key breast cancer-related proteins and favorable pharmacokinetic properties. These results suggest that *A. cardamomum* could serve as a potential source for developing new plant-based therapies against breast cancer. Further *in vitro* and *in vivo* investigations are warranted to validate their efficacy and safety.

## 1. Introduction

*B*reast cancer is one of the most common cancers in women worldwide and a leading cause of cancer-related deaths ( Ye et al., 2023). According to the World Health Organization (WHO), over 2.3 million new cases of breast cancer were diagnosed globally in 2024 ( World Health Organization, 2024). It remains a significant public health challenge due to its high incidence and mortality rates, particularly in low- and middle-income countries with limited access to early detection and advanced treatments ( Francies et al., 2020; Manson & Achel, 2023). Despite advancements in therapeutic strategies such as chemotherapy, targeted therapy, and immunotherapy, breast cancer treatment continues to face challenges, including drug resistance, systemic toxicity, and limited efficacy in aggressive or resistant subtypes ( Liu et al., 2024; Ye et al., 2023). These limitations highlight the critical need for novel therapeutic agents with improved safety and efficacy profiles.

*Amomum cardamomum* (Java cardamom), a member of the *Zingiberaceae family,* is a medicinal plant traditionally valued for its antimicrobial, anti-inflammatory, and antioxidant properties. Our previous study demonstrated that A. cardamomum exhibits significant anticancer activity against breast cancer cell lines, including its ability to inhibit cancer cell proliferation and induce apoptosis ( Khairani et al., 2024). These findings provided strong preliminary evidence of *A. cardamomum*’s potential as a therapeutic agent for breast cancer. Building on this foundation, the present study aims to further explore the therapeutic potential of *A. cardamomum* by elucidating the bioactive compounds responsible for its anticancer effects and their underlying mechanisms of action. Using Liquid Chromatography-Mass Spectrometry (LC-MS), we identified and characterized the phytochemicals present in A. cardamom. A network pharmacology approach was employed to identify key protein targets and pathways associated with these compounds to complement this. Molecular docking studies and molecular dynamics simulations assessed these compounds’ binding interactions and stability with their targets. Additionally, ADMET profiling and toxicity assessments were conducted to evaluate the drug-likeness and safety of the identified compounds *.*

This study represents a critical step in advancing the understanding of *A. cardamomum*’s bioactive potential and its application in addressing the challenges of breast cancer therapy. By integrating in vitro findings, compound characterization, and computational approaches, we aim to provide insights that could contribute to developing novel, plant-based therapeutics for breast cancer treatment.

## 2. Methods

This study employed a multi-faceted methodological framework to comprehensively evaluate the therapeutic potential of *A. cardamomum* for cancer treatment. The methods included LC-MS analysis for compound identification, network pharmacology to identify key protein targets, molecular docking and dynamics to analyze ligand-target interactions, and ADMET profiling and toxicity assessments to evaluate drug-likeness and safety. Each method was selected to address specific aspects of the study objectives and to ensure a thorough investigation of *A. cardamomum*’s bioactive compounds.

### 2.1 Experimental studies

**2.1.1 Liquid Chromatography-Mass Spectrometry (LC-MS) analysis**

The ethanol extract of Java cardamom seeds, which already extracted in our previous study, was analyzed using a UPLC BEH C18 column (1.7 μm, 2.1 × 50 mm) on an LC-MS system operating in positive ion mode. A sample volume of 5 μL was injected into the system for each run. The extract was prepared by dissolving 100 μL of the liquid extract in 900 μL of water, vortexed, and filtered through a 0.22 μm Millex filter before injection. The mobile phase consisted of solvent A (water with 0.1% formic acid) and solvent B (acetonitrile with 0.1% formic acid). The separation was performed using gradient elution with the following profile: 95% A and 5% B at 0.4 minutes, gradually transitioning to 100% B over 15 minutes. The composition was held at 100% B for 0.1 minutes before returning to the initial condition of 95% A and 5% B at 15.1 minutes, which was maintained until 20 minutes for re-equilibration. The flow rate was set to 0.4 mL/min. Mass spectrometry was detected using electrospray ionization (ESI) in positive ion mode, with a mass range of 50–1200 m/z. The capillary voltage was set to 0.5 kV, the cone voltage to 21 V, and the source temperature to 500°C ( Abdallah et al., 2023; Kaur et al., 2022).

### 2.2 In-Silico studies

**2.2.1 Protein-protein interaction and protein network construction**

Protein-protein interaction (PPI) networks were constructed by integrating overlapping protein targets associated with breast cancer, utilizing multiple databases for comprehensive analysis. The process involves querying GeneCards and the Chemical Toxicogenomic Database using the search term “breast cancer” while incorporating predicted protein targets of cardamom compounds obtained from SwissTarget Prediction. The intersection of these protein sets is then input into the STRING database, specifically focusing on Homo sapiens with a medium confidence threshold (score > 0.4). The resulting protein network is subsequently imported into Cytoscape, a network visualization software, for in-depth analysis and more representative visualization of the protein interactions ( Hozhabri et al., 2022). This approach enables researchers to elucidate complex relationships between proteins involved in breast cancer and potential therapeutic targets, providing valuable insights into the molecular mechanisms underlying the disease. By leveraging multiple databases and sophisticated visualization tools, scientists can identify key nodes and clusters within the PPI network, potentially uncovering novel therapeutic targets or biomarkers for breast cancer.

**2.2.2 Protein and ligand preparation**

Four selected proteins from the PPI network result were obtained from the PDB website ( https://www.rcsb.org/; accessed November 14 ^th^, 2024) ( Table 1). The Biovia Discovery Studio v.2021 application was used to separate the control ligand from the three-dimensional crystal structure of the target protein. The two-dimensional structure of A. cardamomum’s bioactive compounds was retrieved from the PubChem website in SDF format ( https://pubchem.ncbi.nlm.nih.gov/; accessed November 14 ^th^, 2024). The MOE v2022.02 application was then used to conserve, neutralize, and refine all ligands and proteins to an RMS gradient of 0.001 kcal/mol/Å2 ( Yenny et al., 2024).

**Table 1.** Structural information of protein targets used in molecular docking analysis.

Protein	PDB ID	Control ligand	Active site amino acid residue
Casp3	5IBC	ACE-ASP-GLU-VAL-ASK	TRP206 ARG207 ASN208 ASP211 GLY212 TRP214 GLN217 PHE247 GLU248 SER249 PHE250
SRC	2H8H	Quinazoline	LEU273 GLY274 GLN275 GLY276 CYS277 PHE278 GLY279 GLU280 VAL281 ALA293 ILE294 LYS295 LEU297 MET314 VAL323 LEU325 ILE336 THR338 GLU339 TYR340 MET341 GLY344 SER345 LEU347 ASP348 ASP386 ARG388 ALA390 ASN391 LEU393 ALA403 ASP404 PHE405 LEU407 ALA408 ILE411 TYR416 LYS423 PRO425
TNF-α	2AZ5	6,7-dimethyl-3-[(methyl {2-[methyl ({1-[3-(trifluoromethyl) phenyl]-1h-indol-3-yl} methyl) amino] ethyl} amino) methyl]-4h-chrome-4-one	1:(HIS73 LYS98 SER99 PRO100 CYS101 GLU116 TYR119)2:(GLY68 CYS69 PRO70 ALA96 LYS98 PRO100 CYS101 GLN102 ARG103 LYS112 PRO113 TRP114 TYR115 GLU116 PRO117 ILE118 TYR119)
EGFR	1M17	Erlotinib	PHE699 VAL702 LYS721 LEU723 LYS730 ALA731 GLU734 ILE735 ASP737 GLU738 TYR740 VAL741 ARG812 ASP813 ARG817 ASN818 ASP831 PHE832 GLY833 LEU834 ALA835 LYS836 LEU837 TYR845 GLU848 LYS851 PRO853

**2.2.3 Pharmacokinetic and pharmacodynamic analysis of *A.cardamom*um compounds**

Assessing a compound’s absorption, distribution, metabolism, excretion, and toxicity (ADMET) characteristics requires multiple computational methods. First, the canonical SMILES notation of the compound was obtained from the PubChem Database. The canonical SMILES notation of the compounds was then inserted into the SwissADME server ( http://www.swissadme.ch/index.php; accessed November 15 ^th^, 2024) to determine the ADME (absorption, distribution, metabolism, and excretion) properties. Furthermore, the toxicity predictions of all the compounds were conducted using the ProTox-II server ( http://tox-new.charite.de/protox_II; accessed November 15 ^th^, 2024) in compliance with the toxicity criteria set by the Organisation for Economic Cooperation and Development (OECD). The drug-likeness of the compound was assessed using Lipinski’s rule of five criteria. The compounds were evaluated for their adherence to Lipinski’s principles by submitting their canonical SMILE molecular structures to the Molinspiration Cheminformatics service ( https://www.molinspiration.com/cgi/properties; accessed November 15 ^th^, 2024).

**2.2.4 Molecular docking studies**

The MOE v2022.02 application was used to perform the molecular docking. MOE’s Site Finder tool automatically finds the amino acid sequences of the target protein’s active site ( Table 1). The results of the molecular docking include 2D/3D visualization, Root Mean Square Deviation (RMSD) (Å), and binding affinity (kcal/mol). Compounds are regarded as potent target protein inhibitors if their RMSD value is less than 2Å and their binding affinity is much lower (more negative value) than the control ligand ( Yenny et al., 2024). Additionally, the type and similarity of interaction between the chosen compound and the control ligand in the target protein’s active site were used to visualize and ensure that the compound could act as a specific inhibitor of the target protein ( Rita et al., 2025).

**2.2.5 Molecular dynamic simulation**

The ligand with the lowest binding affinity was further analyzed through molecular dynamics simulation using Yet Another Scientific Artificial Reality Application (YASARA) software ( Krieger & Vriend, 2015). The simulation parameters were set at 25°C temperature, pH 7.4, and 0.9% salt concentration for a duration of 50 nanoseconds. This designated timeframe significantly exceeds the minimum duration required to assess protein-ligand complex stability, which is typically 20 nanoseconds ( Wargasetia et al., 2021). The simulation was executed using the md_run macro program, and the results were analyzed using the md_analyze function.

## 3. Results

### 3.1 Experimental studies

**3.1.1 Liquid Chromatography-Mass Spectrometry (LC-MS) analysis**

The LC-MS analysis of *Amomum cardamomum* seed extract identified 22 unique compounds ( Figure 1, Table 2). These compounds exhibit diverse molecular weights, retention times, and polarities, reflecting the chemical complexity of the extract. The retention times ranged from 0.52 to 17.09 minutes, and molecular weights varied between 113.115 Da (ethyl cyanoacetate) and 369.161 Da (1,2-diphenyl-3-(phenylmethyl)-1H-indene), highlighting the presence of both small and larger molecules.

**Figure 1.**
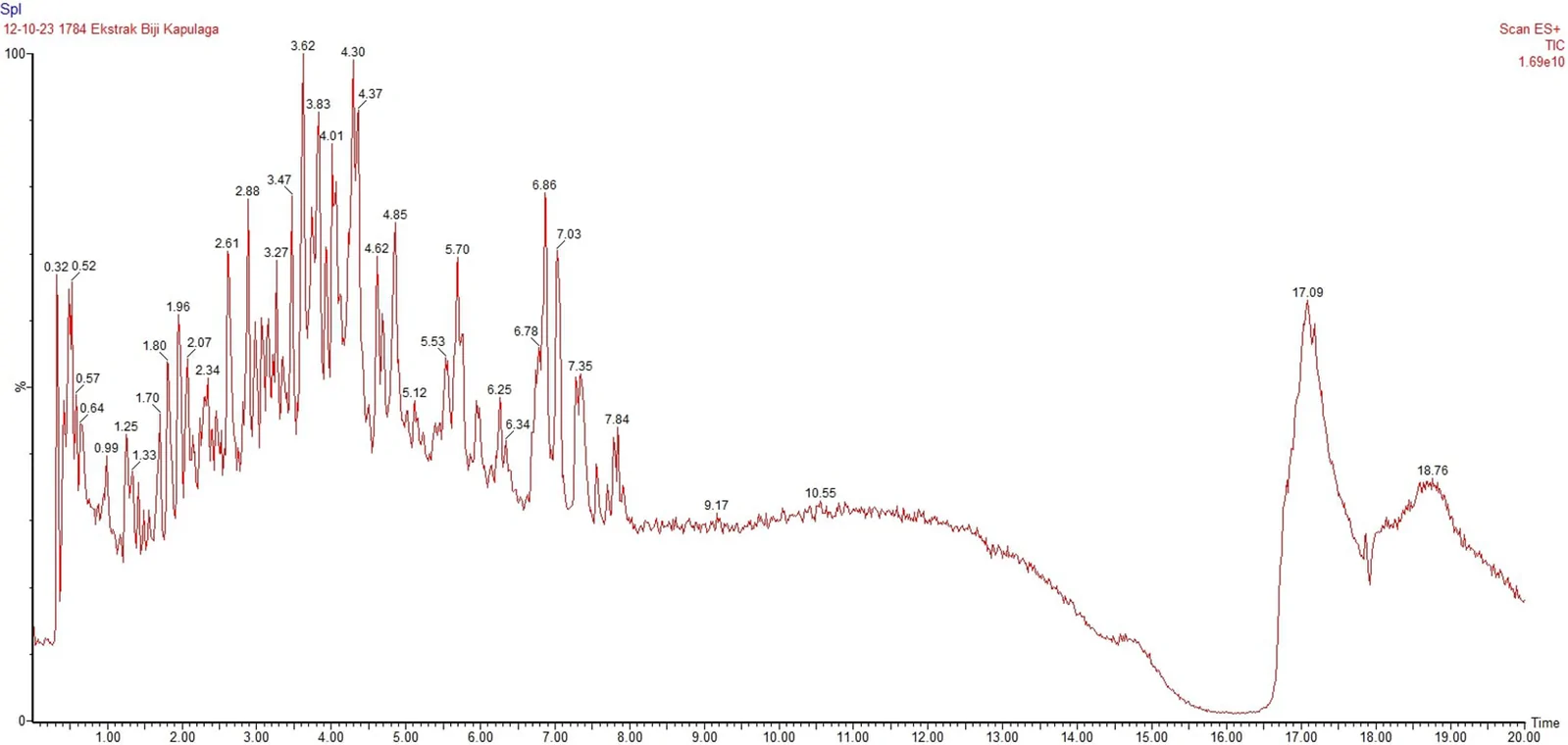
Chromatogram (TIC) of *A. cardamomum* seed extract analyzed by LC-MS.

**Table 2.** List of *A. cardamomum* compounds detected by LC-MS analysis.

No	Name	RT	Parent ion (M/Z)	Molecular formula [M-H] ^+^	Mass molecule (Da)	RT
C1	7-(1,1-Dimethylethyl) 5,6-dihydroimidazo[1,2-a]pyrazine-2,7(8H)-dicarboxylate	0.52	268.1297	C12H17N3O4	267.281	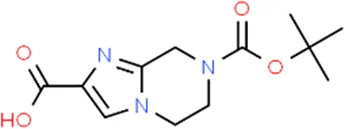
C2	2,4,6-trimethylmelamine	0.994	169.1202	C6H12N6	168.200	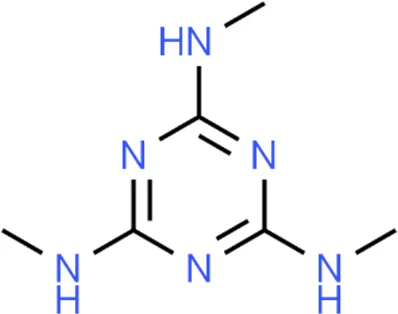
C3	1-Butyl-1H-imidazol 1-Butylimidazole	1.281	123.0949	C7H12N2	124.184	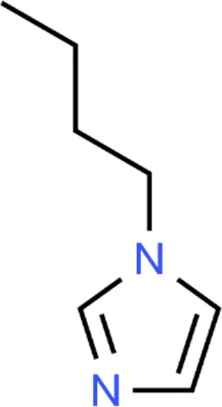
C4	3-(5-Amino-3-methyl-pyrazole-1-yl)-propionitrile	1.702	151.1024	C7H10N4	150.181	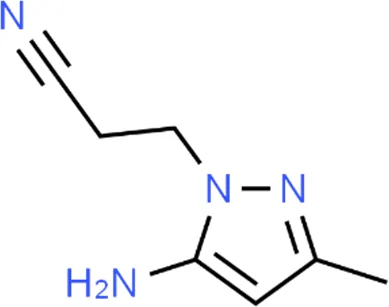
C5	2-[Bis(2-hydroxyethyl)amino]propane-1,3-diol	1.803	180.1216	C7H17NO4	179.214	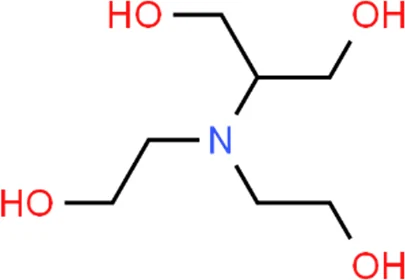
C6	1,2-diphenyl-3-(phenylmethyl)-1H-indene	1.955	369.1614	C28H21	357.466	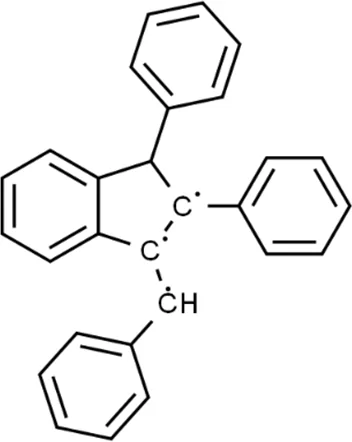
C7	Genipin	2.073	227.0964	C11H14O5	226.226	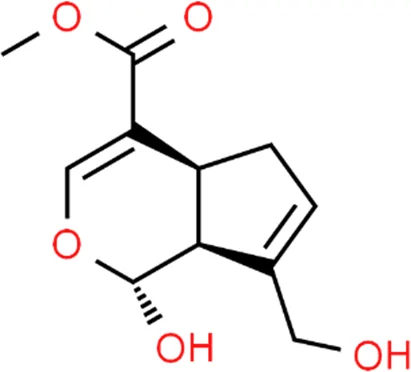
C8	4H,6H-Furo[3,4-c][1,2,5]oxadiazol-4-one	2.343	127.0147	C4H2N2O3	126.070	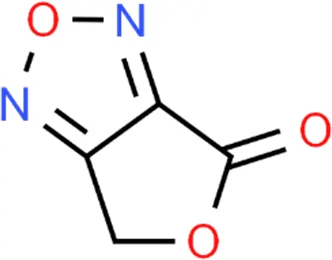
C9	4,6-Dimethyl-5-morpholin-4-ylpyrimidin-2-amine	2.596	209.1409	C10H16N4O	208.260	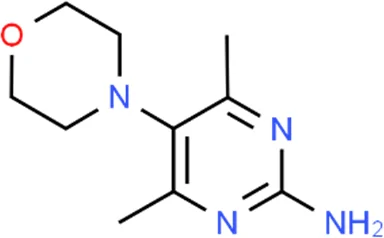
C10	diglyme	2.916	135.1071	C6H14O3	134.174	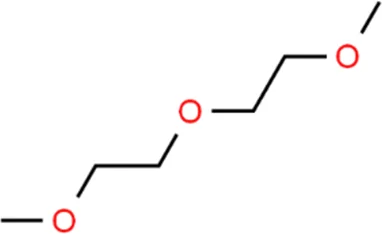
C11	(R)-(+)-Pulegone	3.270	153.1255	C10H16O	152.233	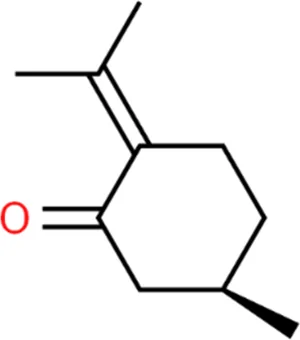
C12	trans-decalin	3.472	139.1534	C10H18	138.250	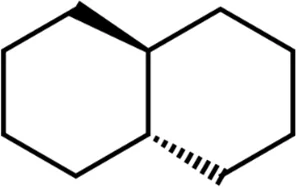
C13	Seratrodast	3.624	355.1887	C22H26O4	354.439	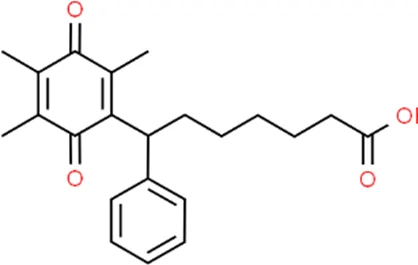
C14	tert-butyl 2,4-dideoxy-3,5-O-(1-methyl ethylidene)-D-erythro-hexonate	3.826	261.1739	C13H24O5	260.327	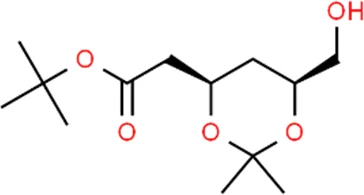
C15	2-Ethyl-N,N-bis(2-ethylhexyl) hexylamine	4.012	340.3940	C23H49N	339.642	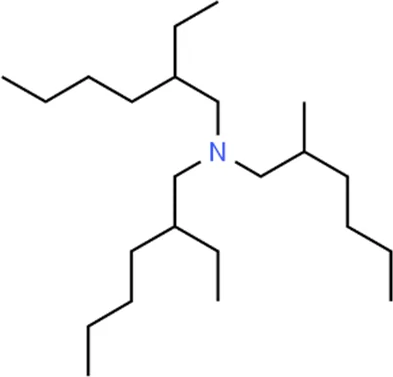
C16	N-Boc-piperidine-3-methanol	4.315	216.1587	C11H21NO3	215.289	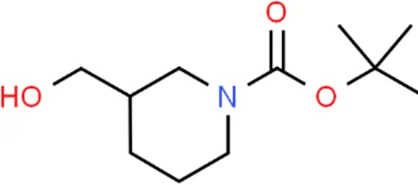
C17	6,10-Dimethyl-1,3,5,7,9,11,12-tridecaneheptol	4.871	325.2201	C15H32O7	324.410	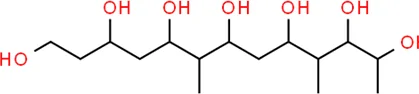
C18	Dihydroxymalonic acid	5.697	137.0670	C3H4O6	136.060	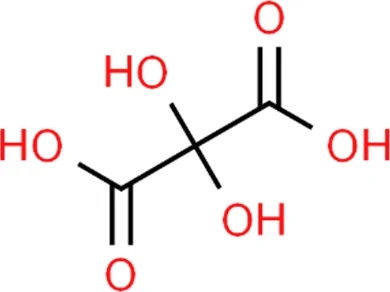
C19	Perillic alcohol	6.254	153.0623	C10H16O	152.233	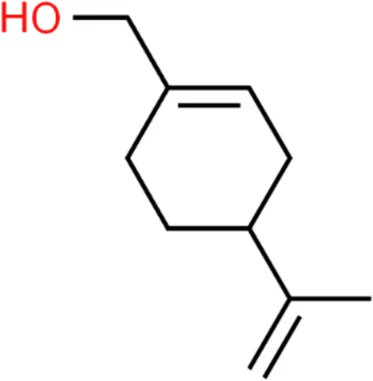
C20	N-[(1-Ethyl-3-methyl-1H-pyrazole-4-yl)methyl]-N-[(1'-methyl-1,4'-bipiperidin-4-yl)methyl]ethanamine	6.860	362.3331	C21H39N5	361.568	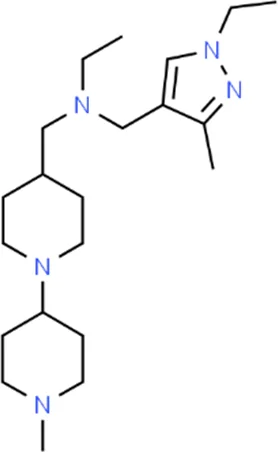
C22	Secoverine	7.838	346.2740	C22H35NO2	345.519	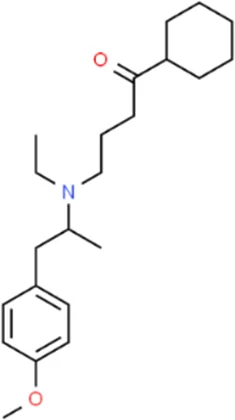
C22	Ethyl cyanoacetate	17.092	114.0541	C5H7NO2	113.115	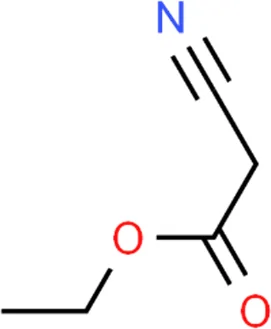

### 3.2. In-silico studies

**3.2.1 Protein-protein interaction network analysis**

The analysis of 22 compounds from cardamom extract revealed 512 potential targets based on Swiss target prediction. These results were compared with breast cancer-related genes from GeneCards (18,182 genes) and CTD (38,877 genes). A Venn diagram showed ( Figure 2.) 450 genes at the intersection of breast cancer and bioactive compounds from *A. cardamomum*, representing the overlap between cardamom-specific targets and breast cancer-related genes.

**Figure 2.**
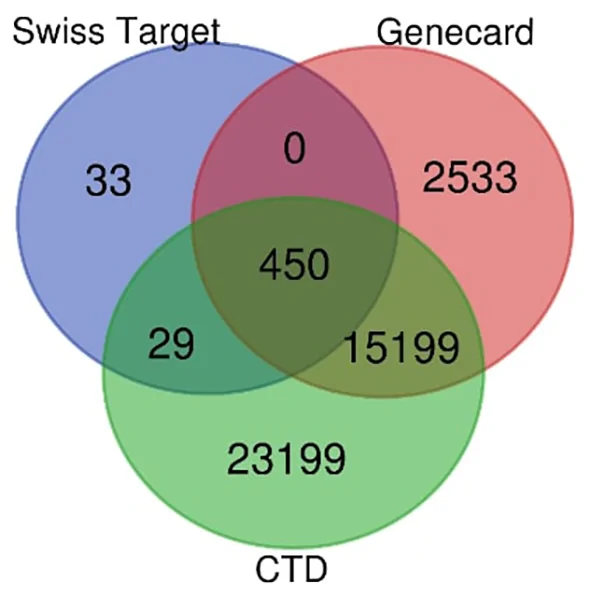
Veen Diagram of active compounds targets of A. cardamomum and breast cancer targets.

To investigate the hub genes within key modules, a protein-protein interaction (PPI) network analysis was conducted using STRING with a confidence score ≥0.4 for the biological species “Homo sapiens” ( Figure 3). This analysis revealed the synergistic and multi-targeted effects of A. compacta, contributing to its potential anticancer properties. As shown in Figure 2, topology analysis using Cytoscape was performed to calculate the centrality of each protein. The study identified CAS3, EGFR, SRC, and TNF-18 as key targets with the highest degree of connectivity, indicating their essential roles in the mechanisms of breast cancer ( Figure 3). These findings are supported by previous research highlighting the importance of these proteins in breast cancer progression and treatment resistance ( Hozhabri et al., 2022).

**Figure 3.**
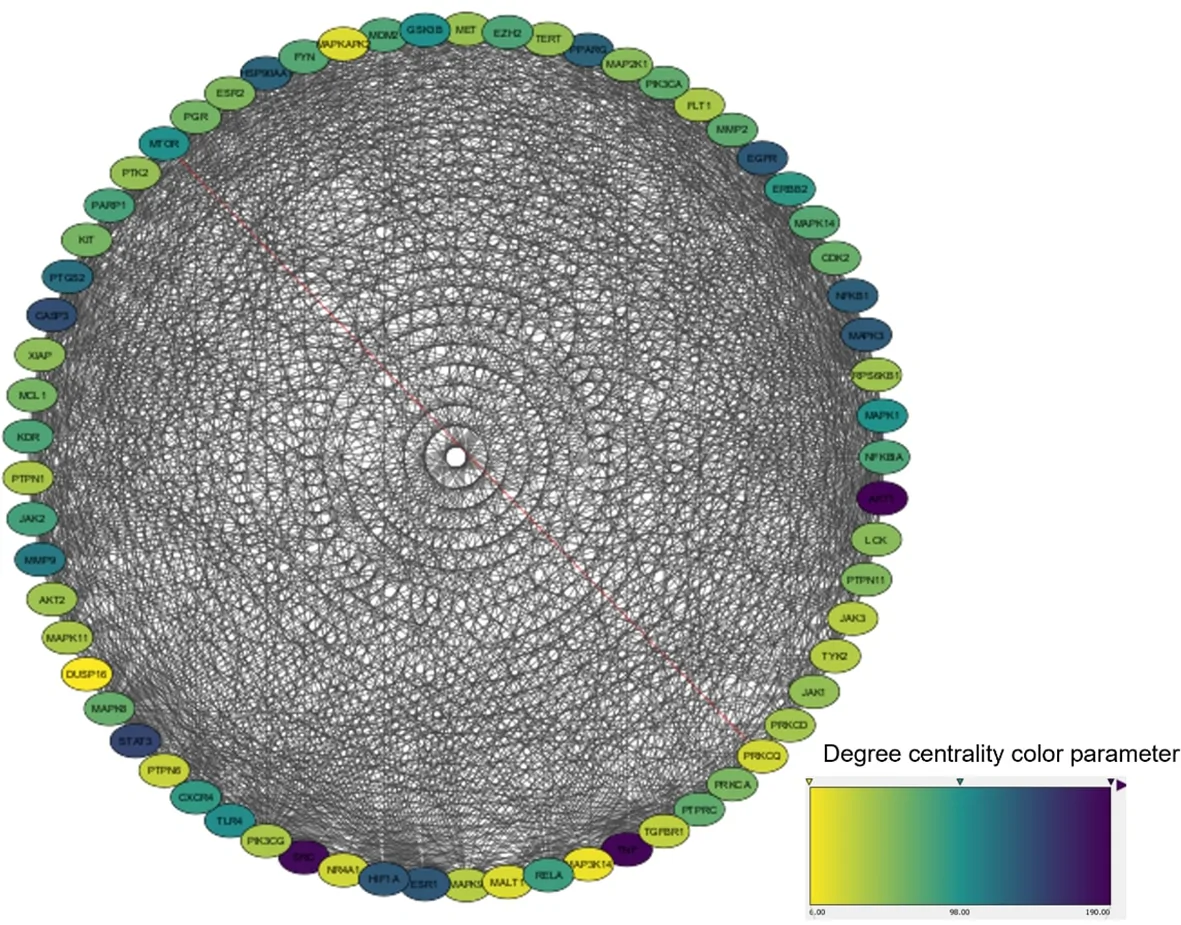
Protein interaction network of A. cardamomum as a target of breast cancer treatment.

The high centrality of these proteins in the PPI network suggests their critical roles in the complex interplay of molecular pathways involved in breast cancer. Their identification as key targets provide valuable insights into potential therapeutic strategies. Consequently, these proteins were selected as ideal candidates for further analysis through molecular docking and molecular dynamics simulations, which can offer a deeper understanding of their interactions with potential anticancer compounds and guide the development of more effective treatments for breast cancer ( Kanhaiya et al., 2017).

**3.2.2 Pharmacokinetic and pharmacodynamic analysis of *A. cardamomum* compound**

The pharmacokinetic and pharmacodynamic analysis of the 22 compounds identified in *A. cardamomum* seed extract revealed several key findings regarding their drug-likeness and potential therapeutic application ( Table 3).

**Table 3.** Absorption, distribution, metabolism, excretion, and toxicity (ADMET) analysis result.

Compound	GI absorption	Human oral bioavailability	BBB permeant	CYP2D6 substrate	CYP2D6 inhibitor	OCT2 Substrate	Log S (ESOL)
C1	97.435 (High)	0.55 (Good)	Yes	No	No	No	-1.95 (Very)
C2	94.257 (High)	0.55 (Good)	No	No	No	No	-1.64 (Very)
C3	95.289 (High)	0.55 (Good)	Yes	No	No	No	-1.58 (Very)
C4	96.632 (High)	0.55 (Good)	No	No	No	No	-0.92 (Very)
C5	56.29 (Low)	0.55 (Good)	No	No	No	No	1.03 (Highly)
C6	100 (High)	0.55 (Good)	No	No	No	No	-6.94 (Poorly)
C7	59.431 (Low)	0.56 (Good)	No	No	No	No	-0.57 (Very)
C8	98.73 (High)	0.55 (Good)	No	No	No	No	-0.74 (Very)
C9	100 (High)	0.55 (Good)	No	No	No	No	-0.74 (Very)
C10	100 (High)	0.55 (Good)	No	No	No	No	-0.05 (Very)
C11	96.77 (High)	0.55 (Good)	Yes	No	No	No	-2.72 (Soluble)
C12	94.707 (High)	0.55 (Good)	Yes	No	No	No	-3.61 (Soluble)
C13	93.316 (High)	0.85 (Good)	Yes	No	No	No	-4.43 (Moderately)
C14	94.923 (High)	0.55 (Good)	Yes	No	No	No	-1.77 (Very)
C15	87.773 (Low)	0.55 (Good)	No	Yes	No	Yes	-6.68 (Poorly)
C16	90.877 (High)	0.55 (Good)	Yes	No	No	No	-1.65 (Very)
C17	100 (High)	0.55 (Good)	No	No	No	No	-0.32 (Very)
C18	0 (Low)	0.56 (Good)	No	No	No	No	0.60 (Very)
C19	94.656 (High)	0.55 (Good)	Yes	No	No	No	2.65 (Soluble)
C20	93.554 (High)	0.55 (Good)	Yes	No	No	No	-3.34 (Soluble)
C21	94.249 (High)	0.55 (Good)	Yes	No	Yes	No	-4.70 (Moderately)
C22	100 (High)	0.55 (Good)	No	No	No	No	-0.58 (Very)

**3.2.2.1 Absorption, distribution, metabolism, excretion, and toxicity (ADMET) analysis**

The pharmacokinetic evaluation ( Table 3) revealed that most compounds from *A. cardamomum* seeds were predicted to have good gastrointestinal absorption, suggesting favorable uptake following oral administration. Only a few compounds, including C5, C7, C15, and C18, showed low absorption potential, indicating possible limitations in bioavailability. Consistent with these findings, nearly all compounds exhibited adequate oral bioavailability, reinforcing their potential for systemic circulation. Interestingly, several molecules, such as C1, C3, C11-C14, C16, C19-C21, were also predicted to penetrate the blood–brain barrier, raising the possibility of central nervous system activity.

In terms of metabolism, the majority of compounds were not identified as substrates or inhibitors of cytochrome P450 2D6 (CYP2D6), which reduces the likelihood of extensive enzyme-mediated clearance or drug–drug interactions. Notably, C15 was predicted to act as a substrate, while C21 displayed inhibitory potential, highlighting that these particular molecules may influence or be influenced by drugs processed through CYP2D6.

Excretion analysis indicated that almost all compounds were unlikely to interfere with renal elimination pathways. Only C15 demonstrated potential inhibitory activity toward the organic cation transporter OCT2, suggesting that most *A. cardamomum* constituents are unlikely to alter the renal clearance of co-administered drugs.

Regarding solubility, the majority of compounds showed adequate water solubility, ranging from moderate to very soluble, which is advantageous for formulation and bioavailability. However, two compounds, C6 and C15, were categorized as poorly soluble, which could limit their practical application unless addressed by formulation strategies. Together, these findings suggest that the chemical profile of *A. cardamomum* seeds is largely consistent with drug-likeness criteria, although specific molecules may require optimization to overcome solubility or metabolic limitations.

**3.2.2.2 Lipinski’s rules of five**

Lipinski’s rule of five indicates that 20 of the 22 evaluated compounds meet the criteria for drug-likeness compounds ( Table. 4). However, two compounds, C15 and C18, do not fully adhere to these criteria. C15 exhibits noncompliance due to the consensus log P value >5 (6.964573) and molar refractivity >130 (130.880981), which indicates potential challenges related to excessive lipophilicity and molecular size. LogP value indicates the level of lipophilicity, where high lipophilicity can result in quickly metabolized compounds, low solubility, and poorly absorbed ( Rutkowska et al., 2013). Molar refractivity values within the range of 40-130 suggest that a substance is likely to have good intestinal absorption and oral bioavailability ( Ya’u Ibrahim et al., 2020). Conversely, C18 fails to meet the criteria because of its H-bond receptor count >5 (6) and molar refractivity <40 (17.236599), indicating potential issues with polarity and molecular size. The H-bond acceptor and H-bond donor present in the structure of a therapeutic agent play a vital role in membrane transport, drug-protein interactions, distribution, and aqueous solubility ( Kenny, 2022). This evaluation provides valuable insights into the drug-like potential of the compounds from *A. cardamomum.*

**Table 4.** Lipinski’s rules of five results of *A.cardamomum* compounds.

Compound	Molecular weight (D) (g/mol)	Consensus log P	H-bond acceptor	H-bond donor	Molar refractory	Violation of Lipinski’s rules
**Standard**	**<500**	**≤ 5**	**<5**	**≤10**	**40-130**	**≤1**
C1	267	0.397590	4	2	64.743385	0
C2	168	-0.109530	3	3	42.125099	0
C3	124	1.178840	0	0	37.558395	0
C4	150	0.142820	2	2	39.077797	0
C5	179	0.843730	4	4	47.034191	0
C6	357	5.262740	0	0	105.343987	1
C7	226	1.603700	5	2	54.122093	1
C8	126	-0.663430	3	0	17.109299	0
C9	208	0.361710	2	2	54.749691	0
C10	134	1.803360	3	0	35.653496	0
C11	152	2.525599	1	0	50.743492	0
C12	138	2.837899	0	0	52.445992	0
C13	312	-0.053101	6	5	77.145782	1
C14	260	3.034269	5	1	72.196793	1
C15	339	6.964573	0	0	130.880981	2
C16	215	2.270190	3	1	63.245789	0
C17	324	3.838249	7	1	89.061790	1
C18	136	-0.715910	6	2	17.236599	2
C19	152	2.232710	1	1	50.070793	0
C20	361	3.902099	0	0	119.441383	0
C21	345	5.108690	2	0	112.781982	1
C22	113	0.992430	3	0	26.986998	1

**3.2.2.3 Toxicity prediction**

Based on the results of the toxicity analysis ( Table 5), three compounds, namely C6, C7, and C14, were found to be in the toxic level (class I-III). Overall, one compound (C14) is classified as class two toxicity (fatal by ingestion (5 < LD50 ≤ 50)), two compounds (C6 and C7) are classified as class III toxicity (toxic by ingestion (50 < LD50 ≤ 300)), twelve compounds (C1, C2, C4, C5, C8, C9, C11, C13, C15, C16, C20, and C21) are classified as class IV toxicity (harmful if swallowed (300 < LD50 ≤ 2000)), six compounds (C3, C10, C17, C18, C19, and C22) are classified as class V toxicity (may be harmful if swallowed (2000 < LD50 ≤ 5000)), and one compound (C12) are classified as class VI toxicity (non-toxic (LD50 > 5000)).

**Table 5.** Toxicity level and organ target toxicity prediction of *A.cardamomum* compounds.

Compound	Predicted LD _50_	Predicted toxicity class	Average similarity	Organ toxicity				
				Hepatotoxicity	Carcinogenicity	Immunotoxicity	Mutagenicity	Cytotoxicity
C1	500 mg/kg	Four (harmful)	46.86%	Inactive	Inactive	Inactive	Inactive	Inactive
C2	350 mg/kg	Four (harmful)	100%	Active	Inactive	Inactive	Inactive	Inactive
C3	3500 mg/kg	Five (may be harmful)	68.6%	Inactive	Inactive	Inactive	Inactive	Inactive
C4	1300 mg/kg	Four (harmful)	46.57%	Inactive	Active	Inactive	Active	Inactive
C5	3900 mg/kg	Four (harmful)	82.22%	Inactive	Active	Inactive	Inactive	Inactive
C6	175 mg/kg	Three (toxic)	68.93%	Inactive	Active	Inactive	Inactive	Inactive
C7	237 mg/kg	Three (toxic)	100%	Inactive	Inactive	Inactive	Inactive	Inactive
C8	1484 mg/kg	Four (harmful)	44.01%	Inactive	Active	Inactive	Active	Inactive
C9	800 mg/kg	Four (harmful)	42.2%	Inactive	Inactive	Inactive	Inactive	Inactive
C10	4970 mg/kg	Five (may be harmful)	100%	Inactive	Active	Inactive	Inactive	Inactive
C11	470 mg/kg	Four (harmful)	100%	Inactive	Inactive	Inactive	Inactive	Inactive
C12	15380 mg/kg	Six (non-toxic)	100%	Inactive	Active	Inactive	Inactive	Inactive
C13	1520 mg/kg	Four (harmful)	100%	Inactive	Inactive	Inactive	Inactive	Inactive
C14	44 mg/kg	Two (fatal)	80.22%	Inactive	Inactive	Inactive	Inactive	Inactive
C15	1400 mg/kg	Four (harmful)	100%	Inactive	Inactive	Inactive	Inactive	Inactive
C16	1000 mg/kg	Four (harmful)	64.91%	Inactive	Inactive	Inactive	Inactive	Inactive
C17	3000 mg/kg	Five (may be harmful)	100%	Inactive	Inactive	Inactive	Inactive	Inactive
C18	2450 mg/kg	Five (may be harmful)	84.86%	Inactive	Inactive	Inactive	Inactive	Inactive
C19	2100 mg/kg	Five (may be harmful)	100%	Inactive	Inactive	Inactive	Inactive	Inactive
C20	500 mg/kg	Four (harmful)	51.4%	Inactive	Inactive	Inactive	Inactive	Inactive
C21	369 mg/kg	Four (harmful)	100%	Inactive	Inactive	Inactive	Inactive	Inactive
C22	4960 mg/kg	Five (may be harmful)	100%	Inactive	Active	Inactive	Inactive	Inactive

**Table 6.** Molecular docking results of SRC, TNF-α, Casp3, and EGFR protein.

Compound	SRC		TNF-α		Casp3		EGFR	
	BA	RMSD	BA	RMSD	BA	RMSD	BA	RMSD
Control	-7.89	1.56	-7.00	1.24	-6.53	1.64	-6.47	1.24
C1	-6.03	1.46	-5.83	2.41	-5.21	1.96	-5.05	1.62
C2	-5.86	0.96	-5.06	1.23	-4.72	1.32	-4.72	0.74
C3	-5.21	1.26	-4.77	1.53	-4.31	0.82	-5.04	0.96
C4	-5.71	0.99	-4.99	1.77	-4.70	0.85	-5.03	1.74
C5	-5.74	1.48	-5.14	1.15	-4.31	1.35	-4.48	1.26
C6	-6.70	1.13	6.32	1.31	-5.22	1.67	-6.02	1.61
C7	-6.37	1.40	-5.45	1.05	-4.46	1.27	-5.29	1.21
C8	-4.72	1.33	-4.26	1.83	-3.87	1.64	-4.51	1.24
C9	-6.38	1.11	-5.17	1.68	-4.76	0.97	-5.00	0.91
C10	-5.47	1.12	-4.70	1.33	-4.37	1.97	-4.90	1.30
C11	-6.13	1.34	-4.87	0.75	-4.18	1.68	-5.37	1.33
C12	-5.27	0.87	-4.45	1.74	-4.12	1.64	-5.00	1.89
C13	-7.27	1.78	-6.20	1.57	-5.90	1.90	-5.23	1.90
C14	-7.09	0.77	-5.67	1.50	-5.42	1.32	-5.47	1.66
C15	-7.83	1.91	-6.72	1.57	-6.02	1.85	-6.03	2.17
C16	-6.94	1.55	-5.65	1.64	-4.91	1.55	-5.26	1.41
C17	-7.84	1.82	-6.91	1.41	-6.70	1.51	-6.20	1.58
C18	-3.65	1.50	-4.31	0.83	-3.98	1.11	-4.19	1.15
C19	-5.55	1.73	-4.99	1.80	-4.55	1.47	-4.48	1.23
C20	-8.58	1.71	-6.88	2.00	-6.29	1.42	-6.43	0.90
C21	-7.70	2.20	-6.62	1.59	-6.34	1.91	-6.38	1.90
C22	-5.15	0.93	-4.47	1.09	-4.05	1.10	-4.52	0.76

Based on the analysis of organ toxicity, only 1 out of 22 compounds (C2) was reported to be active in the hepatotoxicity category. In the next category, 7 compounds (C4, C5, C6, C8, C10, C12, and C22) were reported as active carcinogenicity. Two out of 22 compounds (C4 and C8) were reportedly active in the mutagenicity. All evaluated compounds did not exhibit immunotoxicity or cytotoxicity. This is a positive sign that these compounds may not always risk genetic or cell damage, even if they are considered detrimental or possibly hazardous based on LD50 values [3]. Therefore, even if the chemicals from *A. cardamomum* have a variety of toxicity profiles, any prospective medicinal or pharmacological application must consider the unique risk factors associated with each component. Moreover, in vivo investigations are necessary to validate these in silico results and offer a more thorough comprehension of the safety and effectiveness of these substances.

**3.2.3 Molecular docking**

The molecular docking analysis was conducted using the MOE application. To carry out a targeted molecular docking analysis, the grid box is accurately placed at the active site of each target protein. The grid box settings in the MOE application’s Site Finder feature were automatically modified according to the amino acid residues corresponding to the natural ligand of each protein. Molecular docking results include binding affinity (measured in kcal/mol), RMSD (Å), and visualization in two and three dimensions. A peptide with an RMSD below 2.5 Å and a significantly higher binding affinity (more negative) than the control ligand may be regarded as a promising inhibitor of the target protein.

**3.2.3.1 Molecular docking of SRC protein**

The molecular docking between SRC and *Amomum cardamomum* revealed that the compounds of *Amomum cardamomum* have RMSD values below 2Å. Based on the binding affinity, C20 has the lowest binding affinity (-8.58 kcal/mol), stronger than the control ligand, with a binding energy of -7.89 kcal/mol ( Table 6). C20 formed three interactions: one acidic hydrophilic (Asp404) and two greasy hydrophobic interactions (Ala390 and Phe405). Control ligand formed three interactions: one acidic hydrophilic (Asp404), one basic hydrophilic (Arg388), and one greasy hydrophobic interaction (Val281). These results showed that C20 interacts with Asp404, the same amino acid residue with which the control ligand interacts ( Table 7). It can be concluded that C20 has the potential to be a new specific competitive inhibitor of SRC active site.

**Table 7.** Molecular docking result of SRC protein.

Ligand	2D Interaction	3D Interaction
**Control**	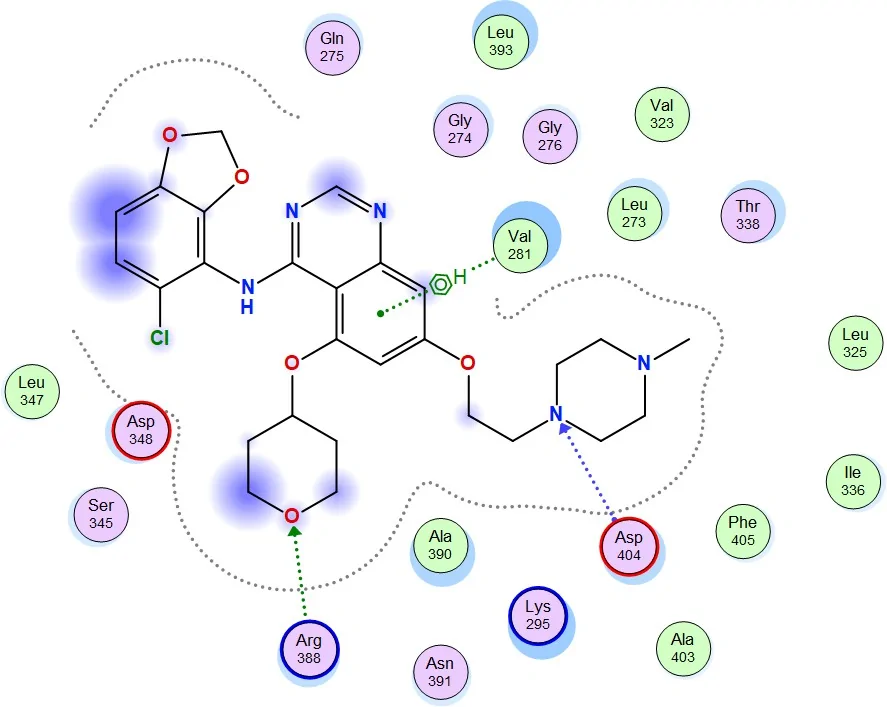	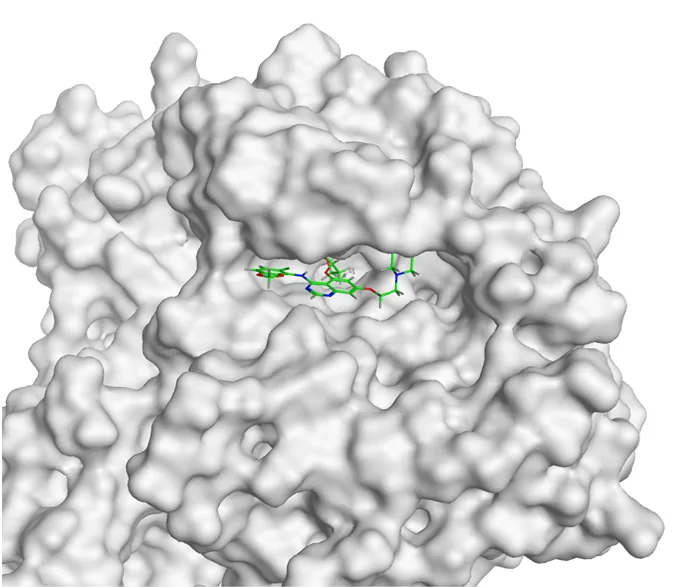
**C20**	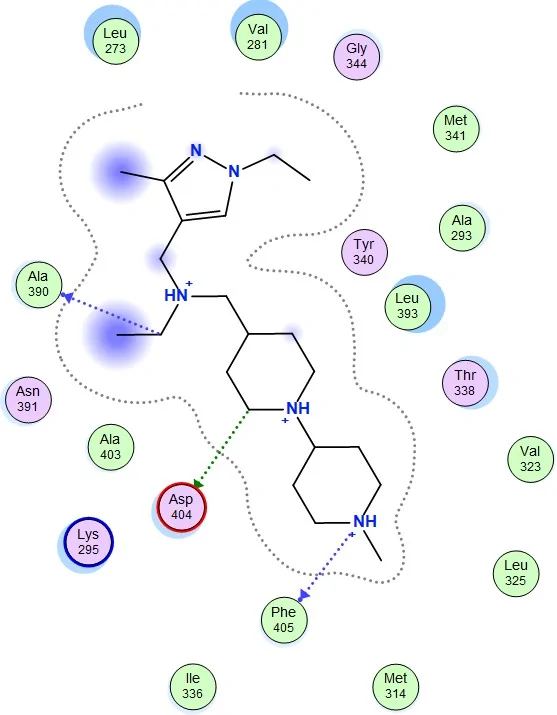	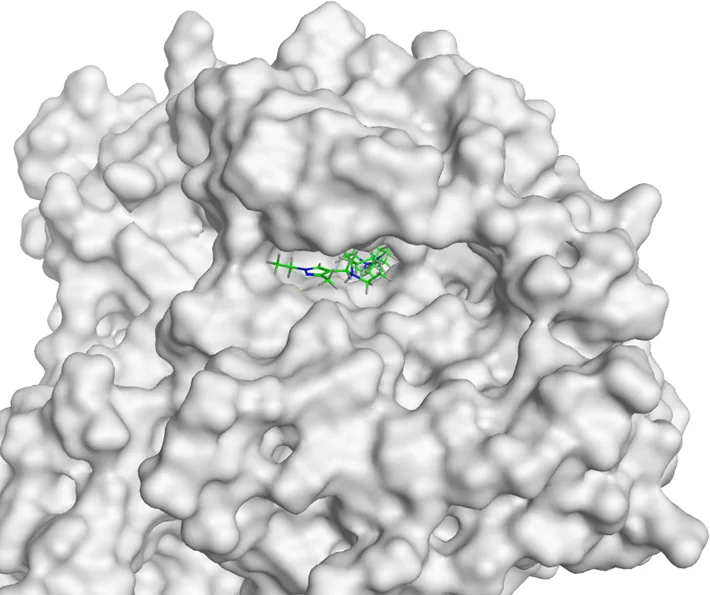

**3.2.3.2 Molecular docking of TNF-α protein**

From the molecular docking result, C17 attaches to the TNF-α active site with minimal deviation, marked by RMSD < 2Å (1.41 Å). C17 (-6.91 kcal/mol) has a binding affinity close to the control ligand’s (-7.00 kcal/mol) ( Table 6). C17 formed three polar hydrophilic interactions: TyrC119, TyrA119 and TyrA151. Meanwhile, the control ligand formed two polar hydrophilic interactions: TyrA119 and TyrA151. These showed that C17 and the control ligand interact with two similar amino acid residues of TNF-α (TyrA119 and TyrA151) ( Table 8). So, C17 has the potential as a new specific competitive inhibitor of TNF-α.

**Table 8.** Molecular docking result of TNF-α protein.

Ligand	2D Interaction	3D Interaction
**Control**	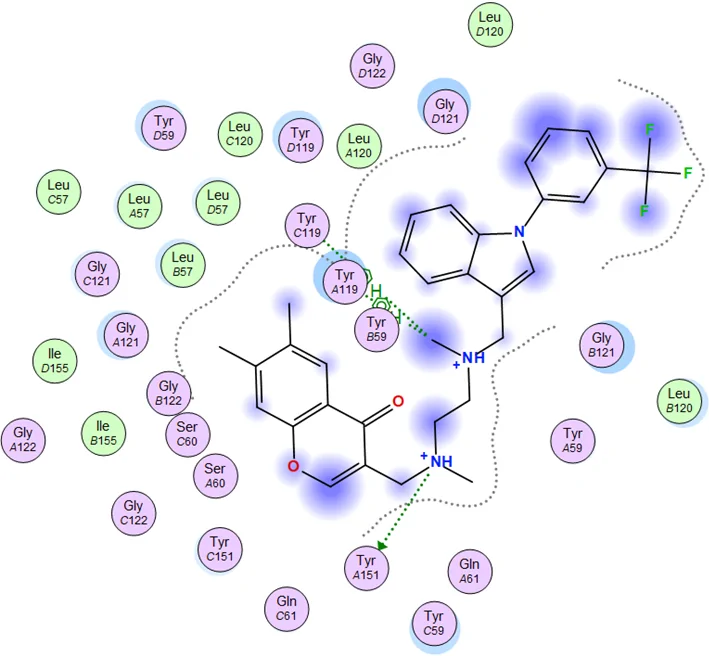	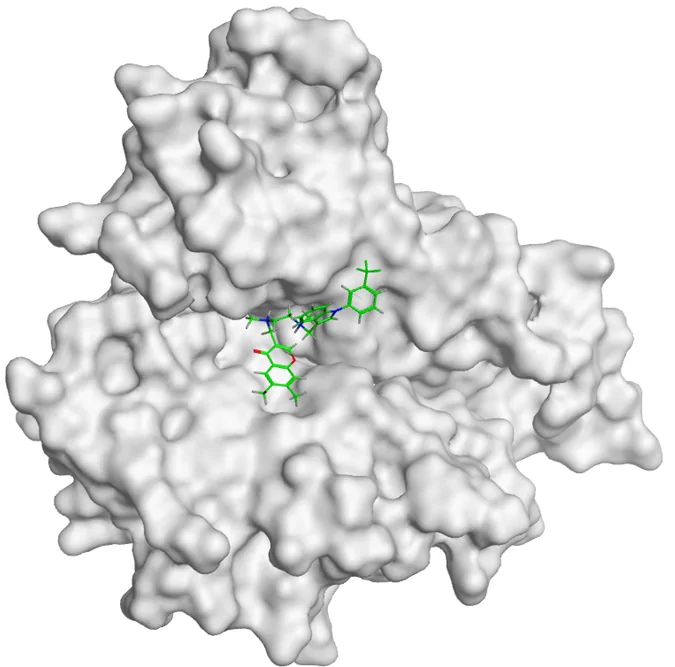
**C17**	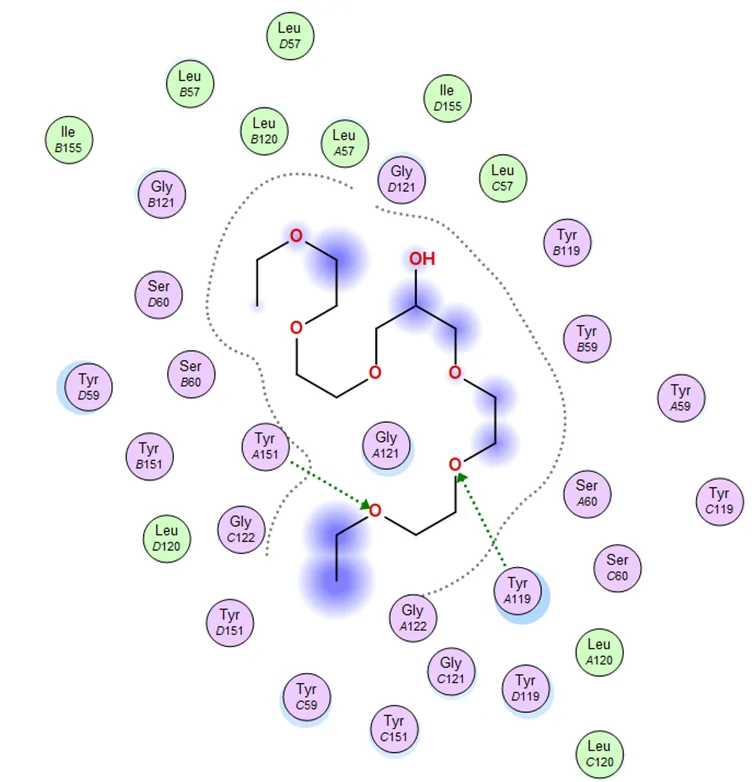	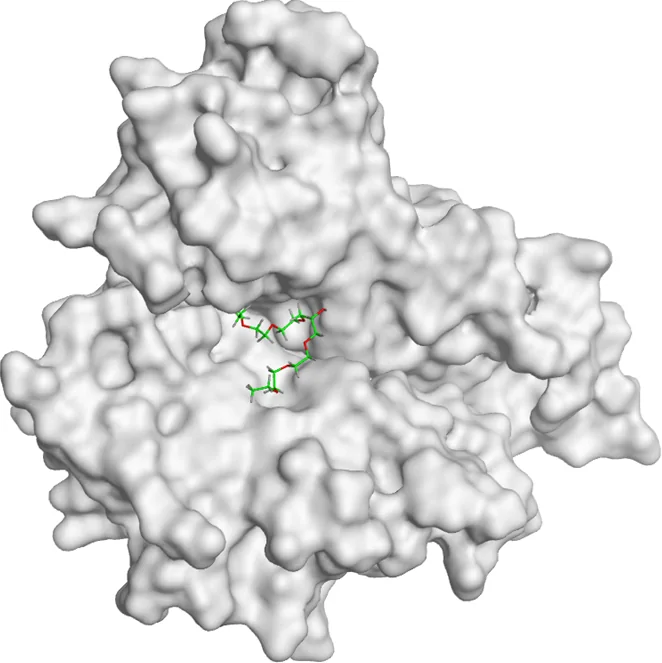

**3.2.3.3 Molecular docking of Casp3 protein**

All compounds of *Amomum cardamomum* have an RMSD value < 2Å. Based on the binding affinity, C17 has the highest binding affinity than the other compounds of *A. (*-6.70 kcal/mol) *cardamomum* and drug control ( Table 6). The 2D interaction visualization revealed that C17 formed three interactions with the active site of casp3: one basic hydrophilic (Arg207), one polar hydrophilic (Asn208), and one greasy hydrophobic (Trp214) interaction. On the other hand, casp3 inhibitors formed one basic hydrophilic (Arg207) and two polar hydrophilic interactions (Thr62, Ser209) with the active site of casp3. This result showed that C17 and casp3 inhibitors formed one similar interaction with the active site of casp3 (Arg207) ( Table 9). In conclusion, C17 has the potential as a new specific competitive inhibitor of the casp3 active site.

**Table 9.** Molecular docking results of casp3 protein.

Ligand	2D Interaction	3D Interaction
**Control Drug**	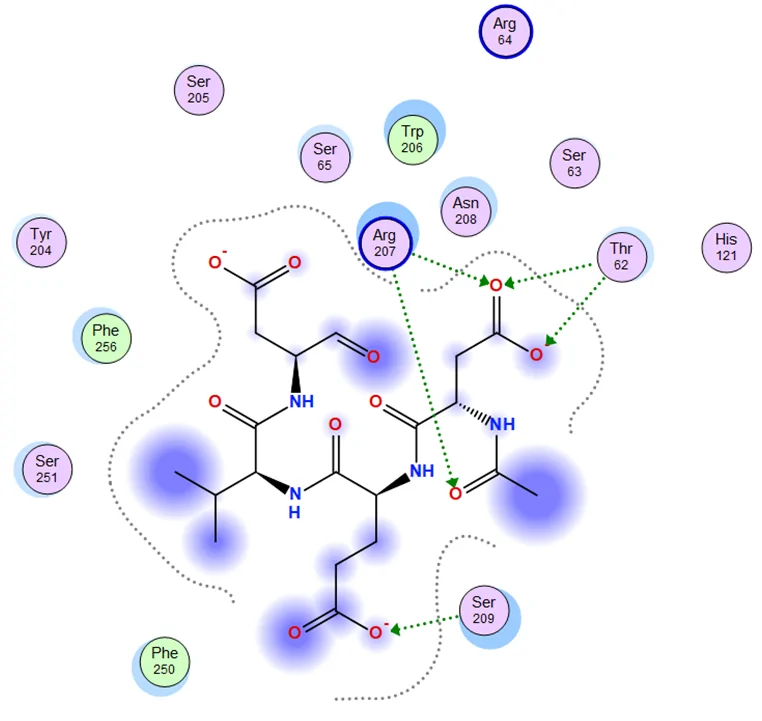	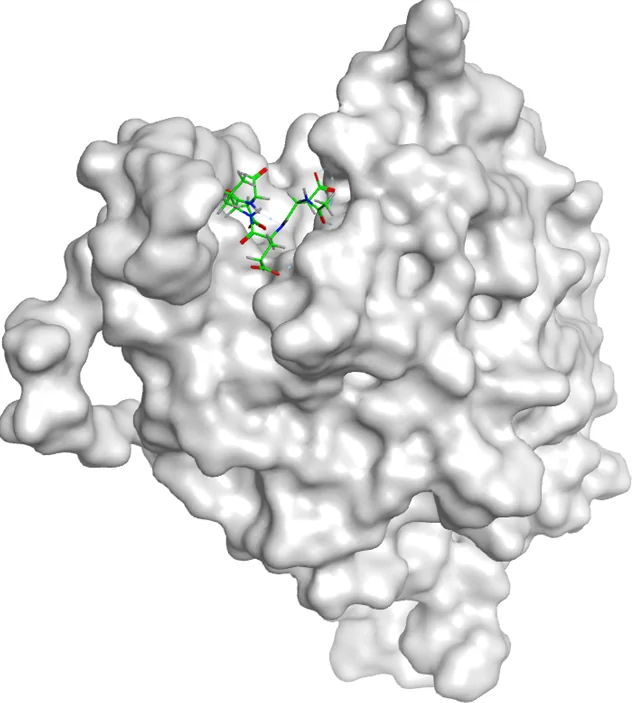
**C17**	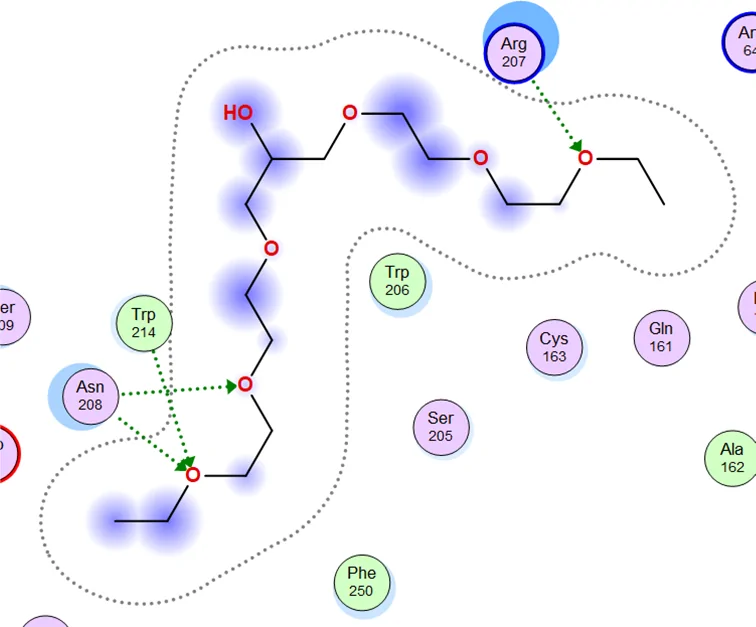	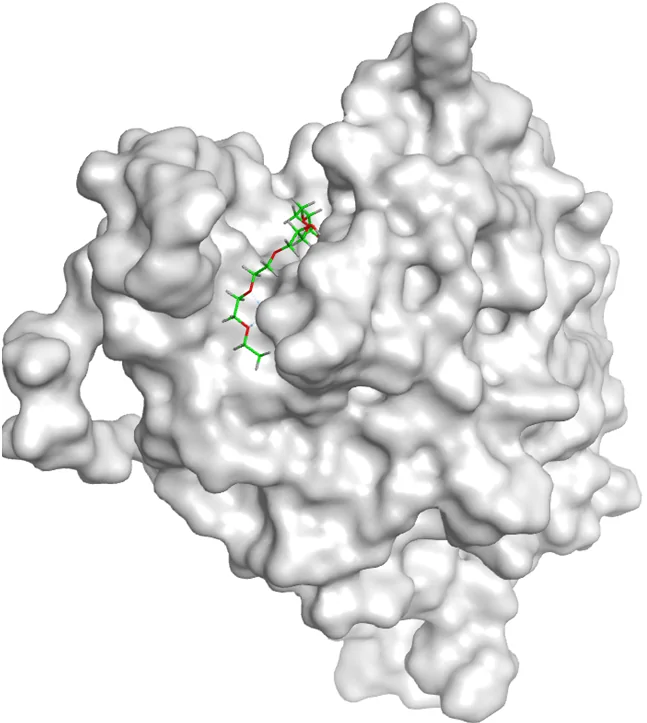

**3.2.3.4 Molecular docking of EGFR protein**

All compounds of *Amomum cardamomum* have RMSD values below 2 Å, except C15. Regarding binding affinity, C20 (-6.43 kcal/mol) has a binding affinity close to the control ligand (-6.47 kcal/mol) as the control ligand ( Table 6). Although the binding affinity of C20 is not stronger than the control ligand, the difference in binding affinity is insignificant. The result of 2D interaction visualization showed that C21 formed two acidic hydrophilic interactions (Asp831 and Glu 738) with the active site of EGFR. Meanwhile, the control ligand formed one basic hydrophilic interaction (Lys721) and one greasy hydrophobic interaction (Phe699). Although C20 does not have identical amino acid residues as the control ligand, the 3D interaction visualization results show that C20 can occupy the binding site of the control ligand on the EGFR active site ( Table 10).

**Table 10.** Molecular docking result of EGFR protein.

Ligand	2D Interaction	3D Interaction
**Control**	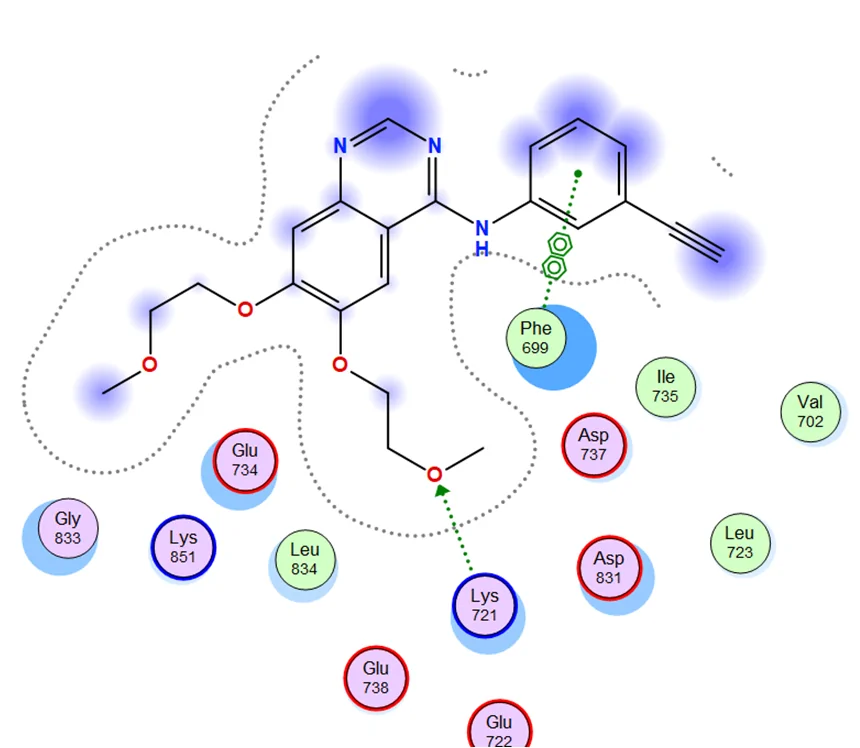	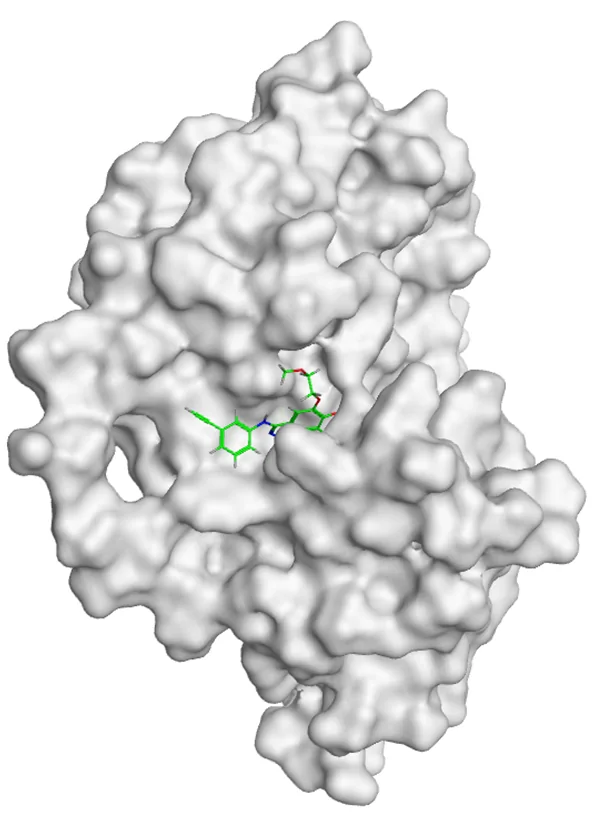
**C20**	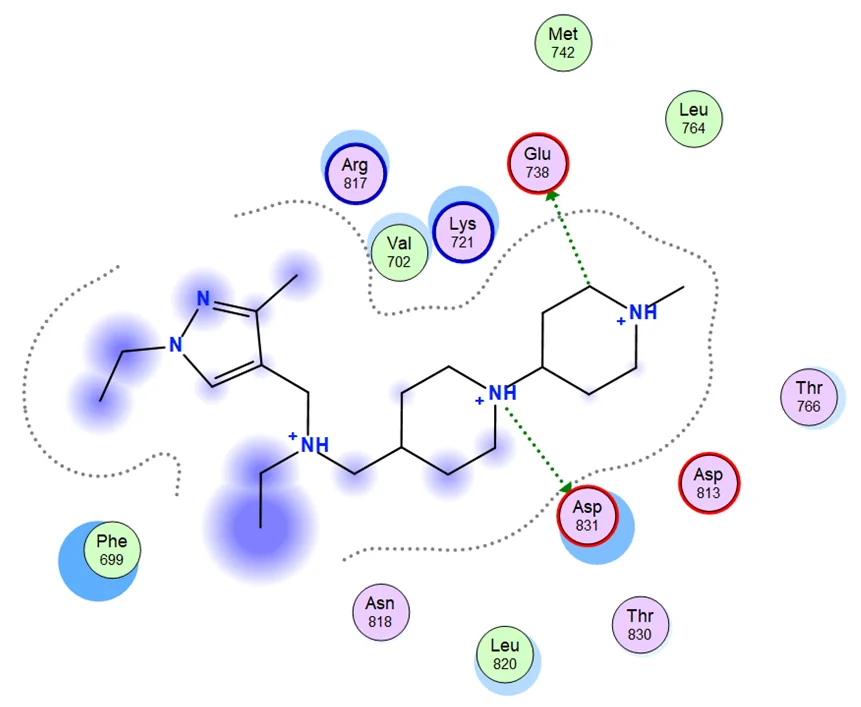	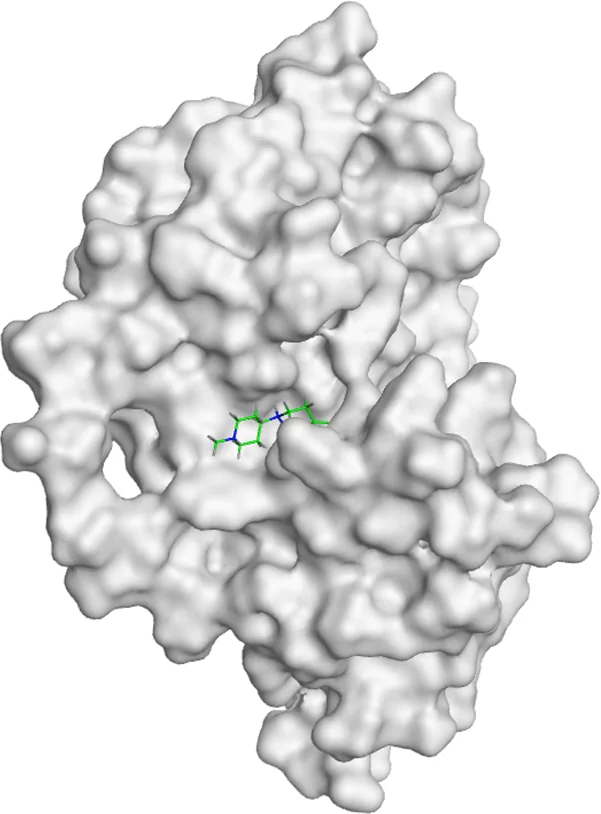

**3.2.4 Molecular dynamic simulation**

The graph illustrates that TNFα-C18 exhibits stable behavior, with RMSD values consistently around 1.5-2.0 Angstroms during the 50 nanoseconds simulation. SRC-C21 shows similar stability with RMSD fluctuating around 1.5-2.0 Angstroms, though with slightly more variation than TNFα-C18. EGFR-C21 demonstrates higher RMSD values (2.5-4.0 Angstroms) with significant fluctuations, particularly in the first 25 nanoseconds, before stabilizing around 2.5 Angstroms. CASP3-C18 shows the highest average RMSD values, maintaining levels between 3.0-3.5 Angstroms throughout the simulation with notable fluctuations. All molecular complexes reach relative stability after approximately 25 nanoseconds, though with different characteristic RMSD ranges ( Figure 4).

**Figure 4.**
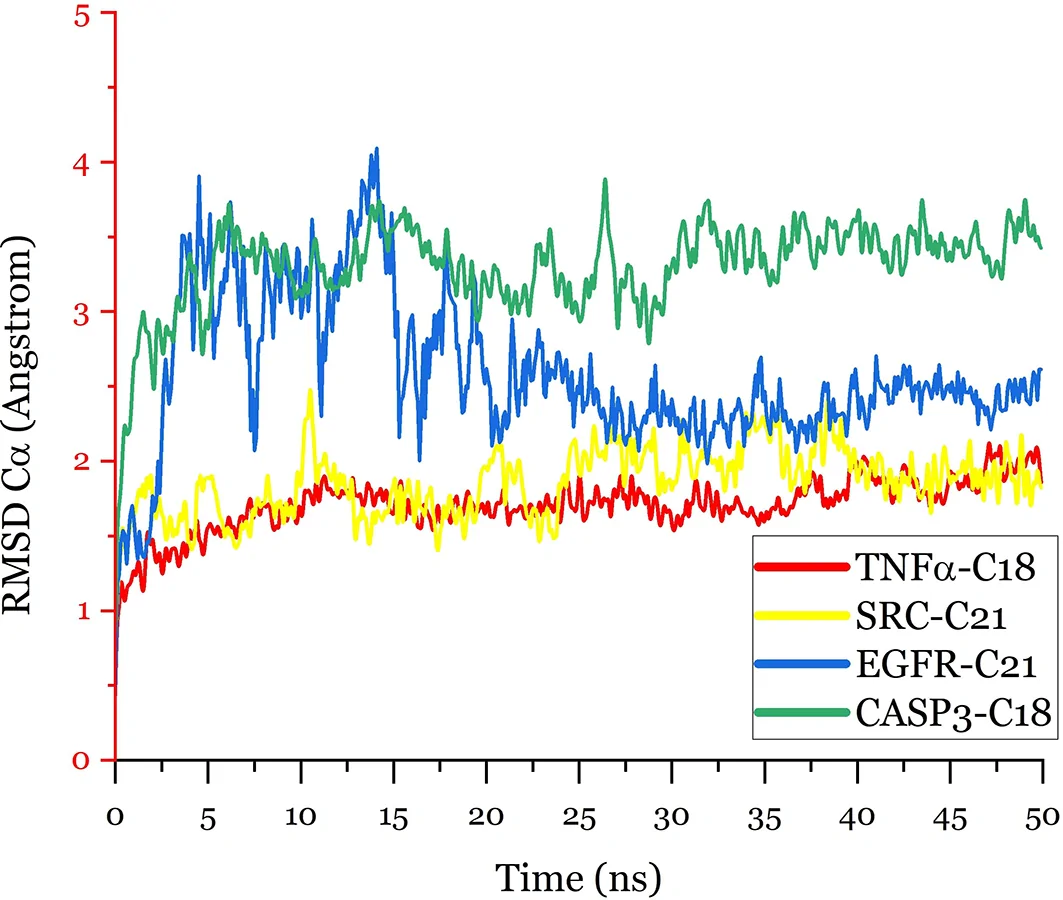
Root Mean Square Deviation (RMSD) trajectories of protein–ligand complexes during 50 ns molecular dynamics simulation.

## 4. Discussion

This study demonstrated the therapeutic potential of *A. cardamomum* through a combination of LC-MS analysis, pharmacokinetic and pharmacodynamic prediction, network pharmacology, molecular docking, and molecular dynamic simulation. LC-MS analysis identified 22 unique compounds in the seed extract, showcasing a chemically diverse profile with various molecular weights and retention times. The protein-protein interaction (PPI) network studies revealed key protein targets for *A. cardamomum* compounds, including SRC, EGFR, TNF-α, and Casp3, all of which play critical roles in breast cancer progression.

SRC, a non-receptor tyrosine kinase and the first proto-oncogene identified in mammalian cells plays a crucial role in breast cancer (BC) by promoting tumor initiation, growth, metastasis, and drug resistance ( Luo et al., 2022). SRC is overexpressed or activated in various stages of BC, where it enhances signaling pathways that promote cell proliferation, survival, and motility, contributing to tumorigenesis. SRC activation leads to the phosphorylation of epidermal growth factor receptor (EGFR) ( Finn, 2008; Pelaz & Tabernero, 2022; Raji et al., 2024; Wheeler et al., 2009). Additionally, SRC interacts with HER2, amplifying signaling through pathways like FAK and STAT3, which increase the metastatic potential and contribute to resistance against HER2-targeted therapies ( Raji et al., 2024). Inhibition of SRC has been shown to disrupt these pathways and reduce tumorigenesis, making SRC a critical target for therapeutic intervention in BC. One compound that can inhibit SRC activity is Quinazoline. Quinazoline exerts its effects by binding to the ATP-binding pocket of SRC, leading to the inhibition of its kinase activity. This interaction disrupts signaling pathways essential for tumor cell survival and growth, such as the PI3K-AKT and MAPK pathways ( Emami et al., 2022).

Tumor necrosis factor (TNF-α) is a pro-inflammatory cytokine that plays a significant role in developing and progressing breast cancer. TNF-α is involved in carcinogenesis by activating several signaling pathways, including the TNF receptor 1 (TNFR1) pathway, which leads to the upregulation of oncoproteins such as HBXIP. This activation promotes cell proliferation, survival, and migration, contributing to tumorigenesis. Elevated TNF-α levels are often observed in breast cancer tissues, and its signaling is linked to increased metastasis and drug resistance, making TNF-α a critical factor in BC progression. Inhibition of TNF-α has been shown to reduce cancer cell proliferation, suppress tumor growth, and decrease metastatic potential. Consequently, TNF-α inhibitors are being explored as potential therapeutic agents for BC treatment. Targeting TNF-α makes it possible to disrupt its downstream signaling pathways, including the NF-κB and MAPK pathways, crucial for maintaining tumor cell survival and inducing inflammatory responses that support tumor growth ( Cai et al., 2017; Mercogliano et al., 2020). Given the importance of TNF-α in breast cancer progression, targeting its activity offers a promising strategy to mitigate carcinogenesis and enhance the efficacy of existing therapies.

Caspase-3 (Casp3) plays a complex role in breast cancer, acting as a key apoptosis regulator, but its dysregulation can contribute to tumor progression and chemotherapy resistance ( Pu et al., 2017). High Casp3 expression is associated with poor prognosis, particularly in HER2-positive tumors and specific patient populations, while Casp3 deficiency reduces tumorigenesis in preclinical models ( Pu et al., 2017; Zhu et al., 2024). Casp3 activation is crucial for apoptosis, but its activity can be inhibited by factors such as the X-linked inhibitor of apoptosis protein (XIAP), which prevents its activation and subsequent cell death ( Yosefzon et al., 2018). These findings highlight Casp3’s dual role in promoting tumorigenesis and resistance to treatment, suggesting it could be a target for therapeutic strategies in breast cancer.

Epidermal growth factor receptor (EGFR) is a key player in breast cancer progression, as it regulates critical processes like cell proliferation, survival, migration, and differentiation. EGFR overexpression in breast cancer leads to the dysregulation of signaling pathways such as PI3K-AKT and MAPK, promoting tumorigenesis, metastasis, and resistance to apoptosis ( Sigismund et al., 2017). This is particularly evident in triple-negative breast cancer (TNBC), where EGFR overexpression is linked to poor prognosis. EGFR also interacts with other receptors, like HER2, amplifying tumor growth signals ( Masuda et al., 2012). Due to its central role, EGFR is potentially to be a target for therapeutic strategies

## 5. Conclusion

The integration of molecular docking and dynamic simulation analyses provided robust evidence for the therapeutic potential of *A. cardamomum* compounds, mainly C17 and C20, in targeting critical proteins involved in breast cancer. These compounds demonstrated strong binding affinities and maintained stable interactions with their targets during simulations, underscoring their potential as multi-targeted agents. However, further experimental validation is necessary to confirm these computational findings and evaluate the efficacy of these compounds in preclinical models. Pharmacokinetic and pharmacodynamic profiling revealed that most compounds exhibited favorable absorption, distribution, metabolism, and excretion (ADMET) properties, with 20 out of 22 compounds classified as highly absorbable and demonstrating good oral bioavailability. Notably, compounds such as C17 and C20 displayed the ability to cross the blood-brain barrier, suggesting potential utility in targeting breast cancer metastases to the central nervous system.

## Ethics approval

Ethics and consent were not required.

## Data Availability

Zenodo: Exploration of *Amomum cardamomum* Seed-Derived Compounds: LC-MS Profiling, Network Pharmacology, and Computational Analyses for Innovative Multi-Targeted Breast Cancer Therapy. Doi: https://doi.org/10.5281/zenodo.17299396 ( Cuandra, 2025). This project contains the following underlying data:
- TNFA-KAPULAGA.xlsx - CASP3-KAPULAGA.xlsx - EGFR-KAPULAGA.xlsx - SRC-KAPULAGA.xlsx Data are available under the terms of the Creative Commons Attribution 4.0 International license (CC-BY 4.0).
