# Exploration of Compounds Identified from *Amomum cardamomum* Seed Extract: LC-MS Profiling, Network Pharmacology, and Computational Analyses for Innovative Multi-Targeted Breast Cancer Therapy

**DOI:** 10.12688/f1000research.168214.2

**Published:** 2026-08-06

**Authors:** Dessy Arisanty, Salsabila Putri Khairani, Kevin Nathaniel Cuandra, Aldi Tamara Rahman, Christopher Daniel Tristan, Angela Stephanie Yahono, Muhammad Samudra Ilham, Ajib Zaim Alamsyah, Bezalel Tioleyson Sarjono, Daivan Febri Juan Setiya, Phelia Klarissa Huang, Alya Jihan Hafidza, Afiq Triyandi Hakam, Muhammad Farid Hamka, Daffa Zachary Rasendriya, Muhammad Naufal Hibatullah, Kuni Zakiyyah Sumargo

**Affiliations:** 1 Biochemistry, Universitas Andalas Faculty of Medicine, Padang, West Sumatra, Indonesia; 2 Biomedical Science, Universitas Andalas Faculty of Medicine, Padang, West Sumatra, Indonesia; 3 Medicine, Universitas Andalas Faculty of Medicine, Padang, West Sumatra, Indonesia; 4 Obstetrics and Gynecology, Cipto Mangunkusumo Hospital, Jakarta, Special Capital Region of Jakarta, Indonesia; 5 Medicine, Universitas Sebelas Maret Faculty of Medicine, Surakarta, Central Java, Indonesia; 6 Medicine, Universitas Gadjah Mada Faculty of Medicine Public Health and Nursing, Sleman, Special Region of Yogyakarta, Indonesia; 7 Biology, Universitas Andalas Faculty of Mathematics and Natural Sciences, Padang, West Sumatra, Indonesia; 8 Medicine, Universitas Udayana Faculty of Medicine, Denpasar, Bali, Indonesia; 9 Medicine, Universitas Sam Ratulangi Faculty of Medicine, Manado, North Sulawesi, Indonesia; 10 Medicine, Universitas Islam Indonesia Faculty of Medicine, Sleman, Special Region of Yogyakarta, Indonesia; 11 Nutritional Science, Universitas Pembangunan Nasional Veteran Jakarta Faculty of Health Science, Jakarta, Special Capital Region of Jakarta, Indonesia; 12 Medicine, Universitas Sumatera Utara Faculty of Medicine, Medan, North Sumatra, Indonesia; 13 Medicine, Universitas Jember Faculty of Medicine, Jember, East Java, Indonesia; 14 Medicine, Universitas Negeri Gorontalo Faculty of Medicine, Gorontalo City, Gorontalo, Indonesia

**Keywords:** Amomum cardamomum, breast cancer, LC-MS, network pharmacology, molecular docking, drug discovery

## Abstract

**Background:**

Breast cancer remains one of the leading causes of cancer-related mortality worldwide, and the emergence of drug resistance, systemic toxicity, and limited efficacy of current therapies highlight the need for safer and more effective treatment. Natural products have emerged as promising sources of multi-target anticancer agents. *A. cardamomum* has demonstrated preliminary anticancer potential, yet the bioactive constituents and their molecular mechanisms in breast cancer remain poorly elucidated.

**Methods:**

This study integrated *in silico* approaches to investigate the therapeutic potential of *A. cardamomum* seed extract against breast cancer. LC–MS analysis identified phytochemical compounds, followed by network pharmacology to determine their potential targets and molecular pathways. Pharmacokinetic and toxicity predictions were assessed through ADMET and Lipinski’s rule of five analyses to evaluate drug-likeness and safety. Molecular docking and molecular dynamics (MD) simulations were conducted to evaluate binding affinity and structural stability of compounds with key oncogenic proteins.

**Results:**

LC-MS profiling identified 22 distinct compounds in *A. cardamomum* seeds. ADMET and Lipinski analyses demonstrated that most compounds possessed high gastrointestinal absorption, favorable oral bioavailability, and low toxicity risk. Network pharmacology highlighting SRC, TNF-α, Caspase-3, and EGFR as central nodes in the protein-protein interaction network. Molecular docking identified compounds C17 and C20 as the most promising bioactives, showing strong binding affinities and interactions similar to control ligands. MD simulations confirmed their stable complexes, indicating conformational stability and robust ligand–protein interactions.

**Conclusion:**

This study highlights the promising multi-target anticancer potential of *A. cardamomum* seeds. Compounds C17 and C20 were identified as lead candidates with strong and stable interactions with key breast cancer-related proteins and favorable pharmacokinetic properties. These results suggest that *A. cardamomum* could serve as a potential source for developing new plant-based therapies against breast cancer. Further *in vitro* and *in vivo* investigations are warranted to validate their efficacy and safety.

## 1. Introduction

Breast cancer is one of the most common cancers in women worldwide and a leading cause of cancer-related deaths ( Ye et al., 2023). According to the World Health Organization (WHO), over 2.3 million new cases of breast cancer were diagnosed globally in 2024 ( World Health Organization, 2024). It remains a significant public health challenge due to its high incidence and mortality rates, particularly in low- and middle-income countries with limited access to early detection and advanced treatments ( Francies et al., 2020; Manson & Achel, 2023). Despite advancements in therapeutic strategies such as chemotherapy, targeted therapy, and immunotherapy, breast cancer treatment continues to face challenges, including drug resistance, systemic toxicity, and limited efficacy in aggressive or resistant subtypes ( Liu et al., 2024; Ye et al., 2023). These limitations highlight the critical need for novel therapeutic agents with improved safety and efficacy profiles.

*Amomum cardamomum* (Java cardamom), a member of the *Zingiberaceae family,* is a medicinal plant traditionally valued for its antimicrobial, anti-inflammatory, and antioxidant properties. Our previous study demonstrated that A. cardamomum exhibits significant anticancer activity against breast cancer cell lines, including its ability to inhibit cancer cell proliferation and induce apoptosis ( Khairani et al., 2024). These findings provided strong preliminary evidence of *A. cardamomum*’s potential as a therapeutic agent for breast cancer. Building on this foundation, the present study aims to further explore the therapeutic potential of *A. cardamomum* by elucidating the bioactive compounds responsible for its anticancer effects and their underlying mechanisms of action. Using Liquid Chromatography-Mass Spectrometry (LC-MS), we identified and characterized the phytochemicals present in A. cardamom. A network pharmacology approach was employed to identify key protein targets and pathways associated with these compounds to complement this. Molecular docking studies and molecular dynamics simulations assessed these compounds’ binding interactions and stability with their targets. Additionally, ADMET profiling and toxicity assessments were conducted to evaluate the drug-likeness and safety of the identified compounds *.*

This study represents a critical step in advancing the understanding of *A. cardamomum*’s bioactive potential and its application in addressing the challenges of breast cancer therapy. By integrating in vitro findings, compound characterization, and computational approaches, we aim to provide insights that could contribute to developing novel, plant-based therapeutics for breast cancer treatment.

## 2. Methods

This study employed a multi-faceted methodological framework to comprehensively evaluate the therapeutic potential of *A. cardamomum* for cancer treatment. The methods included LC-MS analysis for compound identification, network pharmacology to identify key protein targets, molecular docking and dynamics to analyze ligand-target interactions, and ADMET profiling and toxicity assessments to evaluate drug-likeness and safety. Each method was selected to address specific aspects of the study objectives and to ensure a thorough investigation of *A. cardamomum*’s bioactive compounds.

### 2.1 Experimental studies

**2.1.1 Liquid Chromatography-Mass Spectrometry (LC-MS) analysis of *A. cardamomum* seed extract**

LC-MS analysis was conducted at Laboratorium Penelitian dan Pengujian Terpadu (LPPT), Gadjah Mada University, using an LC-MS system equipped with a UPLC BEH C18 column (1.7 μm, 2.1 × 50 mm) in positive electrospray ionization (ESI+) mode. The mobile phase consisted of water containing 0.1% formic acid (A) and acetonitrile containing 0.1% formic acid (B). Gradient elution was performed at a flow rate of 0.4 mL/min with the following composition: 95% A/5% B at 0.4 min, gradually changed to 100% B at 15 min, returned to the initial condition at 15.1 min, and maintained until 20 min. For sample preparation, 100 μL of liquid extract was diluted with 900 μL distilled water, while paste-form extract was dissolved in distilled water until homogeneous. All samples were vortexed, filtered using a 0.22 μm Millex filter, and injected into the LC-MS system at 5 μL. Mass spectra were acquired in the m/z range of 50–1200 with a cone voltage of 21 V, capillary voltage of 0.5 kV, and source temperature of 500°C ( Abdallah et al., 2023; Kaur et al., 2022).

### 2.2 In-Silico studies

**2.2.1 Protein-protein interaction and protein network construction**

Protein targets of *A. cardamomum* compounds were predicted using the SwissTargetPrediction and Similarity Ensemble Approach (SEA) web servers. Breast cancer-associated genes were retrieved from GeneCards and the Comparative Toxicogenomics Database (CTD) using the keyword “breast cancer.” The overlapping targets between the compound-related and disease-related gene sets were identified using the InteractiVenn web server. The common targets were then imported into STRING (Homo sapiens, confidence score > 0.4) to construct the protein–protein interaction (PPI) network, which was visualized and analyzed in Cytoscape. Hub genes were prioritized using the CytoNCA plugin via network centrality analysis, and nodes were ranked by overall centrality ( Hozhabri et al., 2022).

**2.2.2 Gene ontology and KEGG pathway enrichment analysis**

Functional enrichment analysis was performed to characterize the biological roles and pathways associated with the overlapping targets in breast cancer. The gene set was analyzed in the DAVID web server using Homo sapiens as the reference organism. GO enrichment was assessed for BP, MF, and CC, whereas KEGG analysis was used to identify relevant signaling pathways. Terms and pathways with FDR < 0.05 were considered significant, and the top ten enriched GO and KEGG results were visualized using the SRplot web server.

**2.2.3 Protein and ligand preparation**

Four selected proteins from the PPI network result were obtained from the PDB web servers ( https://www.rcsb.org/; accessed November 14 ^th^, 2024) ( Table 1). The Biovia Discovery Studio v.2021 application was used to separate the control ligand from the three-dimensional crystal structure of the target protein. The two-dimensional structure of A. cardamomum’s bioactive compounds was retrieved from the PubChem website in SDF format ( https://pubchem.ncbi.nlm.nih.gov/; accessed November 14 ^th^, 2024). The MOE v2022.02 application was then used to conserve, neutralize, and refine all ligands and proteins to an RMS gradient of 0.001 kcal/mol/Å2 ( Yenny et al., 2024).

**Table 1.** Structural information of protein targets used in molecular docking analysis.

Protein	PDB ID	Control ligand	Active site amino acid residue
Casp3	5IBC	ACE-ASP-GLU-VAL-ASK	TRP206 ARG207 ASN208 ASP211 GLY212 TRP214 GLN217 PHE247 GLU248 SER249 PHE250
SRC	2H8H	Quinazoline	LEU273 GLY274 GLN275 GLY276 CYS277 PHE278 GLY279 GLU280 VAL281 ALA293 ILE294 LYS295 LEU297 MET314 VAL323 LEU325 ILE336 THR338 GLU339 TYR340 MET341 GLY344 SER345 LEU347 ASP348 ASP386 ARG388 ALA390 ASN391 LEU393 ALA403 ASP404 PHE405 LEU407 ALA408 ILE411 TYR416 LYS423 PRO425
TNF-α	2AZ5	6,7-dimethyl-3-[(methyl {2-[methyl ({1-[3-(trifluoromethyl) phenyl]-1h-indol-3-yl} methyl) amino] ethyl} amino) methyl]-4h-chrome-4-one	1:(HIS73 LYS98 SER99 PRO100 CYS101 GLU116 TYR119)2:(GLY68 CYS69 PRO70 ALA96 LYS98 PRO100 CYS101 GLN102 ARG103 LYS112 PRO113 TRP114 TYR115 GLU116 PRO117 ILE118 TYR119)
EGFR	1M17	Erlotinib	PHE699 VAL702 LYS721 LEU723 LYS730 ALA731 GLU734 ILE735 ASP737 GLU738 TYR740 VAL741 ARG812 ASP813 ARG817 ASN818 ASP831 PHE832 GLY833 LEU834 ALA835 LYS836 LEU837 TYR845 GLU848 LYS851 PRO853

**2.2.4 Pharmacokinetic and pharmacodynamic analysis of *A.cardamom*um compounds**

Assessing a compound’s absorption, distribution, metabolism, excretion, and toxicity (ADMET) characteristics requires multiple computational methods. First, the canonical SMILES notation of the compound was obtained from the PubChem Database. The canonical SMILES notation of the compounds was then inserted into the SwissADME server ( http://www.swissadme.ch/index.php; accessed November 15 ^th^, 2024) to determine the ADME (absorption, distribution, metabolism, and excretion) properties. Furthermore, the toxicity predictions of all the compounds were conducted using the ProTox-II server ( http://tox-new.charite.de/protox_II; accessed November 15 ^th^, 2024) in compliance with the toxicity criteria set by the Organisation for Economic Cooperation and Development (OECD). The drug-likeness of the compound was assessed using Lipinski’s rule of five criteria. The compounds were evaluated for their adherence to Lipinski’s principles by submitting their canonical SMILE molecular structures to the Molinspiration Cheminformatics service ( https://www.molinspiration.com/cgi/properties; accessed November 15 ^th^, 2024).

**2.2.5 Molecular docking studies**

The MOE v2022.02 application was used to perform the molecular docking. MOE’s Site Finder tool automatically finds the amino acid sequences of the target protein’s active site ( Table 1). Native ligands were isolated using BIOVIA Discovery Studio Visualizer version 2024 (Dassault Systèmes BIOVIA, San Diego, CA, USA) and re-docked for validation; all RMSD values were <2.0 Å. The results of the molecular docking include 2D/3D visualization, Root Mean Square Deviation (RMSD) (Å), and binding affinity (kcal/mol). Compounds are regarded as potent target protein inhibitors if their RMSD value is less than 2Å and their binding affinity is much lower (more negative value) than the control ligand ( Yenny et al., 2024). Additionally, the type and similarity of interaction between the chosen compound and the control ligand in the target protein’s active site were used to visualize and ensure that the compound could act as a specific inhibitor of the target protein ( Rita et al., 2025).

**2.2.6 Molecular dynamic simulation**

The ligand with the lowest binding affinity was further analyzed through molecular dynamics simulation using Yet Another Scientific Artificial Reality Application (YASARA) software ( Krieger & Vriend, 2015). The simulation parameters were set at 25°C temperature, pH 7.4, and 0.9% salt concentration for a duration of 50 nanoseconds. This designated timeframe significantly exceeds the minimum duration required to assess protein-ligand complex stability, which is typically 20 nanoseconds ( Wargasetia et al., 2021). The simulation was executed using the md_run macro program, and the results were analyzed using the md_analyze function.

## 3. Results

### 3.1 Experimental studies

**3.1.1 Liquid Chromatography-Mass Spectrometry (LC-MS) analysis**

The LC-MS analysis of *Amomum cardamomum* seed extract identified 22 unique compounds ( Figure 1, Table 2). These compounds exhibit diverse molecular weights, retention times, and polarities, reflecting the chemical complexity of the extract. The retention times ranged from 0.52 to 17.09 minutes, and molecular weights varied between 113.115 Da (ethyl cyanoacetate) and 369.161 Da (1,2-diphenyl-3-(phenylmethyl)-1H-indene), highlighting the presence of both small and larger molecules.

**Figure 1.**
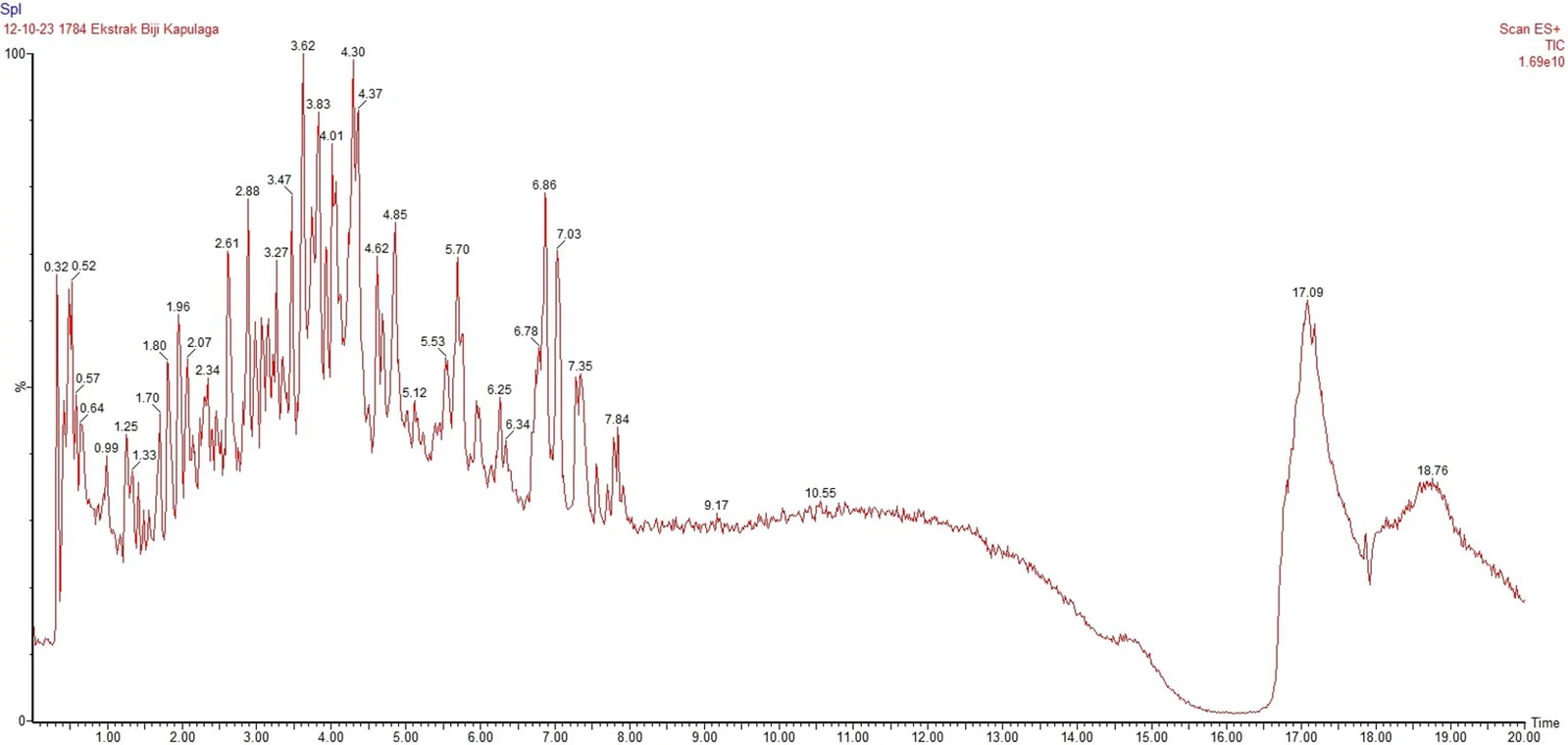
Chromatogram (TIC) of *A. cardamomum* seed extract analyzed by LC-MS.

**Table 2.** List of *A. cardamomum* compounds detected by LC-MS analysis.

No	Name	RT	Parent ion (M/Z)	Molecular formula [M-H] ^+^	Mass molecule (Da)	RT
C1	7-(1,1-Dimethylethyl) 5,6-dihydroimidazo[1,2-a]pyrazine-2,7(8H)-dicarboxylate	0.52	268.1297	C12H17N3O4	267.281	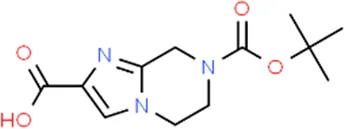
C2	2,4,6-trimethylmelamine	0.994	169.1202	C6H12N6	168.200	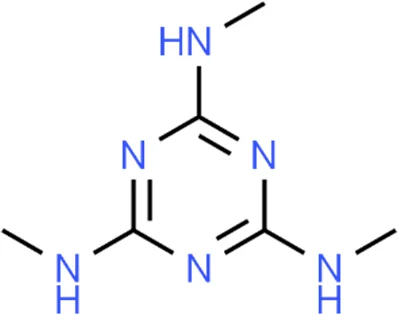
C3	1-Butyl-1H-imidazol 1-Butylimidazole	1.281	123.0949	C7H12N2	124.184	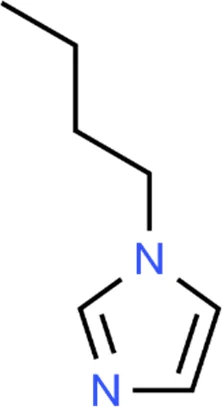
C4	3-(5-Amino-3-methyl-pyrazole-1-yl)-propionitrile	1.702	151.1024	C7H10N4	150.181	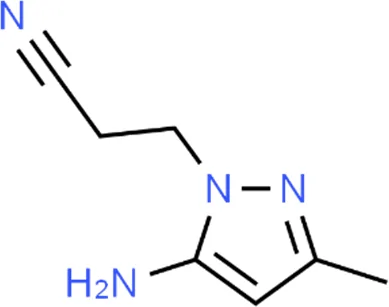
C5	2-[Bis(2-hydroxyethyl)amino]propane-1,3-diol	1.803	180.1216	C7H17NO4	179.214	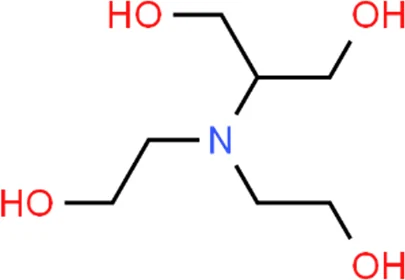
C6	1,2-diphenyl-3-(phenylmethyl)-1H-indene	1.955	369.1614	C28H21	357.466	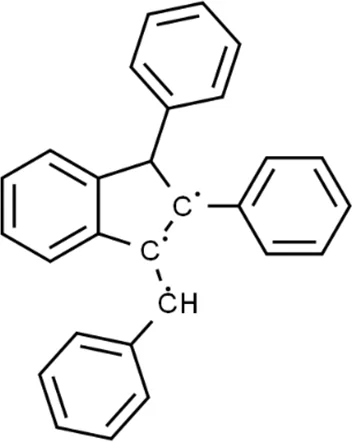
C7	Genipin	2.073	227.0964	C11H14O5	226.226	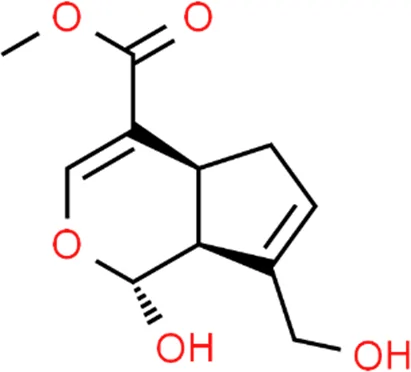
C8	4H,6H-Furo[3,4-c][1,2,5]oxadiazol-4-one	2.343	127.0147	C4H2N2O3	126.070	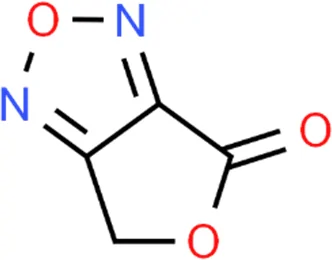
C9	4,6-Dimethyl-5-morpholin-4-ylpyrimidin-2-amine	2.596	209.1409	C10H16N4O	208.260	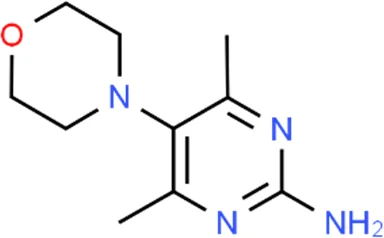
C10	diglyme	2.916	135.1071	C6H14O3	134.174	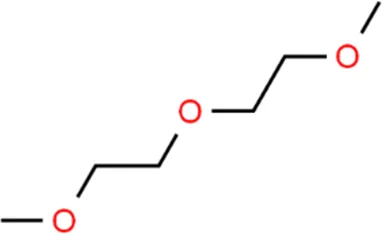
C11	(R)-(+)-Pulegone	3.270	153.1255	C10H16O	152.233	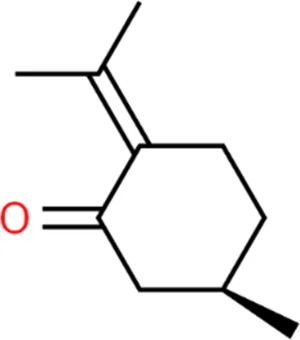
C12	trans-decalin	3.472	139.1534	C10H18	138.250	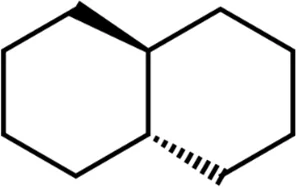
C13	Seratrodast	3.624	355.1887	C22H26O4	354.439	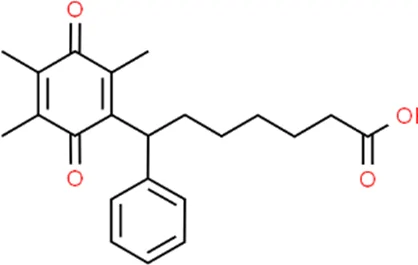
C14	tert-butyl 2,4-dideoxy-3,5-O-(1-methyl ethylidene)-D-erythro-hexonate	3.826	261.1739	C13H24O5	260.327	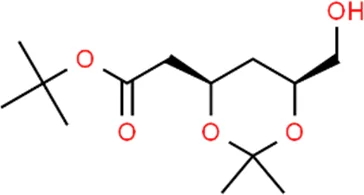
C15	2-Ethyl-N,N-bis(2-ethylhexyl) hexylamine	4.012	340.3940	C23H49N	339.642	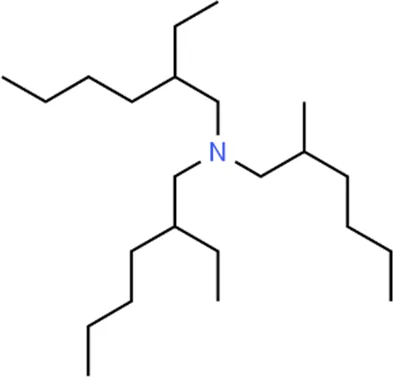
C16	N-Boc-piperidine-3-methanol	4.315	216.1587	C11H21NO3	215.289	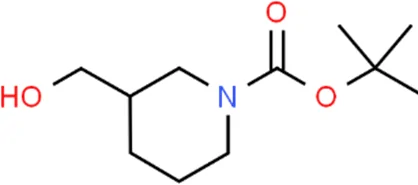
C17	6,10-Dimethyl-1,3,5,7,9,11,12-tridecaneheptol	4.871	325.2201	C15H32O7	324.410	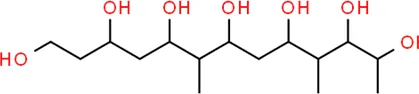
C18	Dihydroxymalonic acid	5.697	137.0670	C3H4O6	136.060	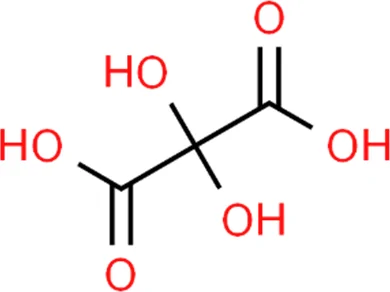
C19	Perillic alcohol	6.254	153.0623	C10H16O	152.233	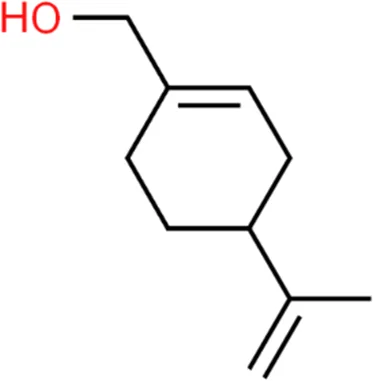
C20	N-[(1-Ethyl-3-methyl-1H-pyrazole-4-yl)methyl]-N-[(1'-methyl-1,4'-bipiperidin-4-yl)methyl]ethanamine	6.860	362.3331	C21H39N5	361.568	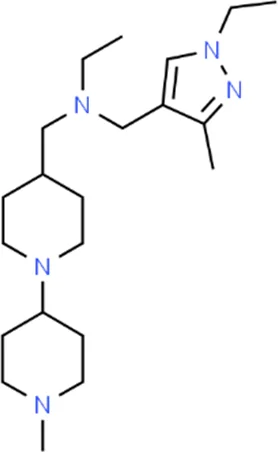
C21	Secoverine	7.838	346.2740	C22H35NO2	345.519	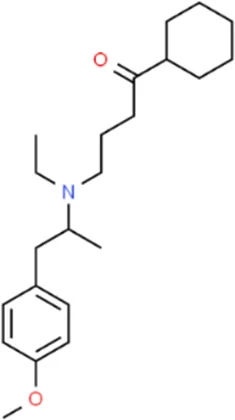
C22	Ethyl cyanoacetate	17.092	114.0541	C5H7NO2	113.115	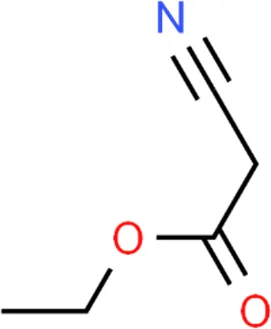

### 3.2 In-silico studies

**3.2.1 Protein-protein interaction network analysis**

A total of 335 protein targets were identified from SwissTargetPrediction, whereas 150 targets were obtained from SEA analysis. Disease-associated gene mining retrieved 20,521 breast cancer-related genes from GeneCards and 41,282 genes from CTD. Venn intersection analysis identified 36 overlapping targets shared between the compound- and disease-related datasets, which were subsequently selected for downstream network analysis ( Figure 2a). The 36 common targets were imported into STRING to construct the protein–protein interaction (PPI) network. The network showed dense interactions among proteins associated with inflammatory signaling, apoptosis, kinase regulation, and matrix remodeling pathways, indicating that the bioactive compounds of *A. cardamomum* may act through multiple interconnected biological mechanisms rather than a single target ( Figure 2b). Several proteins demonstrated high interaction connectivity, particularly TNF, SRC, EGFR, CASP3, PIK3CA, and JAK1, suggesting their important roles in the predicted anti-breast-cancer activity of the identified compounds ( Figure 3c).

**Figure 2.**
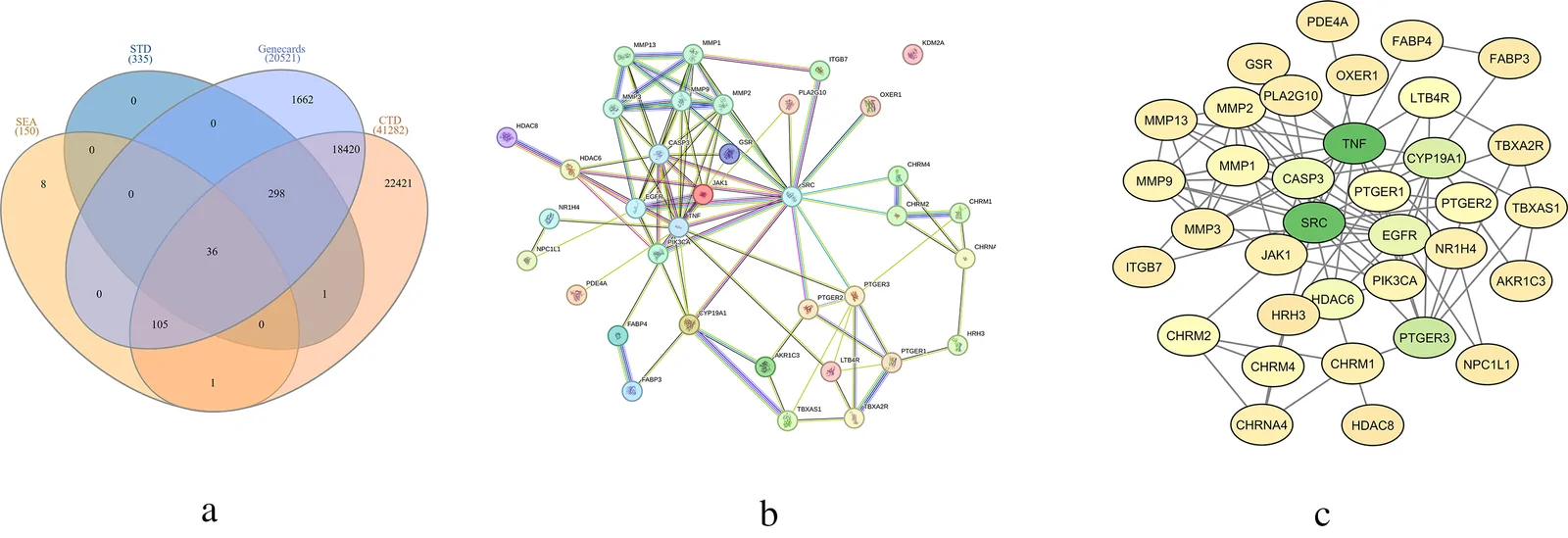
Protein-Protein Interaction Network Analysis. (a) Venn diagram showing the overlap between compound-related and breast cancer-related targets, identifying 36 common genes; (b) PPI network of the overlapping targets; and (c) Topological analysis of PPI network highlighting hub nodes.

**Figure 3.**
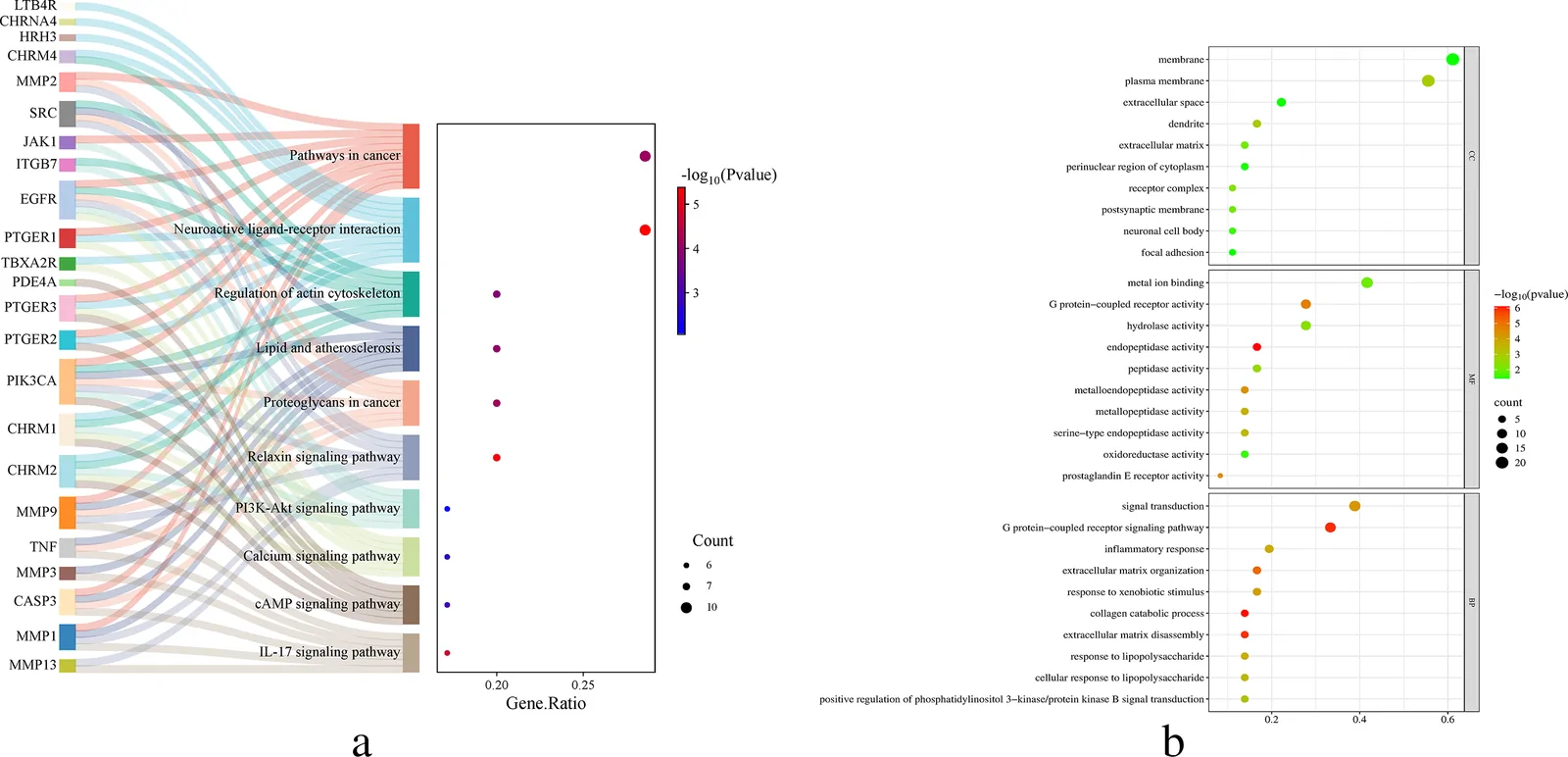
GO and KEGG Enrichment. (a) network depicting association between enriched pathways and genes; and (b) biological process (BP), cellular component (CC), and molecular function (MF)).

Topological analysis using CytoNCA followed by PCA-based overall centrality scoring identified TNF as the highest-ranked hub protein with an overall score of 28.35, followed by SRC (27.13), EGFR (4.39), and CASP3 (2.87) ( Table 3).

**Table 3.** Topological characteristics and overall centrality scores of the prioritized hub proteins identified from the PPI network analysis.

Protein	Betweenness	Closeness	Degree	Eigenvector	Overall
TNF	3.74	2.64	2.97	2.12	28.35
SRC	3.60	2.49	2.50	1.69	27.13
EGFR	0.40	1.33	1.55	1.74	4.39
CASP3	0.14	1.44	1.79	1.95	2.87

**3.2.2 GO and KEGG enrichment**

Functional enrichment analysis demonstrated that the overlapping targets were significantly involved in multiple cancer-related and inflammatory signaling pathways. KEGG pathway analysis revealed that the identified genes were predominantly enriched in pathways associated with cancer, neuroactive ligand–receptor interaction, regulation of the actin cytoskeleton, lipid and atherosclerosis, proteoglycans in cancer, relaxin signaling pathway, PI3K–Akt signaling pathway, calcium signaling pathway, cAMP signaling pathway, and IL-17 signaling pathway. Among these, pathways in cancer and neuroactive ligand–receptor interaction exhibited the highest gene ratios and enrichment significance ( Figure 3a).

As illustrated in Figure 3b, GO enrichment analysis further demonstrated that the shared targets were associated with diverse biological processes, molecular functions, and cellular components. In the BP category, the enriched terms mainly included signal transduction, G protein–coupled receptor signaling pathway, inflammatory response, extracellular matrix organization, collagen catabolic process, and positive regulation of PI3K/AKT signaling. MF analysis showed enrichment in metal ion binding, G protein–coupled receptor activity, hydrolase activity, endopeptidase activity, and oxidoreductase activity. In the CC category, the targets were primarily localized to the membrane, plasma membrane, extracellular space, extracellular matrix, receptor complex, and focal adhesion. Collectively, these findings suggest that the predicted targets may regulate breast cancer progression through coordinated modulation of inflammatory signaling, extracellular matrix remodeling, receptor-mediated communication, and oncogenic PI3K–Akt-related pathways.

**3.2.3 Pharmacokinetic and pharmacodynamic analysis of *A. cardamomum* compound**

The pharmacokinetic and pharmacodynamic analysis of the 22 compounds identified in *A. cardamomum* seed extract revealed several key findings regarding their drug-likeness and potential therapeutic application.

**3.2.3.1 Absorption, distribution, metabolism, excretion, and toxicity (ADMET) analysis**

The pharmacokinetic evaluation ( Table 4) revealed that most compounds from *A. cardamomum* seeds were predicted to have good gastrointestinal absorption, suggesting favorable uptake following oral administration. Only a few compounds, including C5, C7, C15, and C18, showed low absorption potential, indicating possible limitations in bioavailability. Consistent with these findings, nearly all compounds exhibited adequate oral bioavailability, suggested favorable pharmacokinetic properties. Interestingly, several molecules, such as C1, C3, C11-C14, C16, C19-C21, were also predicted to penetrate the blood–brain barrier, raising the possibility of central nervous system activity.

**Table 4.** Absorption, distribution, metabolism, excretion, and toxicity (ADMET) analysis result.

Compound	GI absorption	Human oral bioavailability	BBB permeant	CYP2D6 substrate	CYP2D6 inhibitor	OCT2 Substrate	Log S (ESOL)
C1	97.435 (High)	0.55 (Good)	Yes	No	No	No	-1.95 (Very)
C2	94.257 (High)	0.55 (Good)	No	No	No	No	-1.64 (Very)
C3	95.289 (High)	0.55 (Good)	Yes	No	No	No	-1.58 (Very)
C4	96.632 (High)	0.55 (Good)	No	No	No	No	-0.92 (Very)
C5	56.29 (Low)	0.55 (Good)	No	No	No	No	1.03 (Highly)
C6	100 (High)	0.55 (Good)	No	No	No	No	-6.94 (Poorly)
C7	59.431 (Low)	0.56 (Good)	No	No	No	No	-0.57 (Very)
C8	98.73 (High)	0.55 (Good)	No	No	No	No	-0.74 (Very)
C9	100 (High)	0.55 (Good)	No	No	No	No	-0.74 (Very)
C10	100 (High)	0.55 (Good)	No	No	No	No	-0.05 (Very)
C11	96.77 (High)	0.55 (Good)	Yes	No	No	No	-2.72 (Soluble)
C12	94.707 (High)	0.55 (Good)	Yes	No	No	No	-3.61 (Soluble)
C13	93.316 (High)	0.85 (Good)	Yes	No	No	No	-4.43 (Moderately)
C14	94.923 (High)	0.55 (Good)	Yes	No	No	No	-1.77 (Very)
C15	87.773 (Low)	0.55 (Good)	No	Yes	No	Yes	-6.68 (Poorly)
C16	90.877 (High)	0.55 (Good)	Yes	No	No	No	-1.65 (Very)
C17	100 (High)	0.55 (Good)	No	No	No	No	-0.32 (Very)
C18	0 (Low)	0.56 (Good)	No	No	No	No	0.60 (Very)
C19	94.656 (High)	0.55 (Good)	Yes	No	No	No	2.65 (Soluble)
C20	93.554 (High)	0.55 (Good)	Yes	No	No	No	-3.34 (Soluble)
C21	94.249 (High)	0.55 (Good)	Yes	No	Yes	No	-4.70 (Moderately)
C22	100 (High)	0.55 (Good)	No	No	No	No	-0.58 (Very)

In terms of metabolism, the majority of compounds were not identified as substrates or inhibitors of cytochrome P450 2D6 (CYP2D6), which reduces the likelihood of extensive enzyme-mediated clearance or drug–drug interactions. Notably, C15 was predicted to act as a substrate, while C21 displayed inhibitory potential, highlighting that these particular molecules may influence or be influenced by drugs processed through CYP2D6.

Excretion analysis indicated that almost all compounds were unlikely to interfere with renal elimination pathways. Only C15 was predicted to be an OCT2 substrate, suggesting that most *A. cardamomum* constituents are unlikely to affect the renal clearance of co-administered drugs.

Regarding solubility, the majority of compounds showed adequate water solubility, ranging from moderate to very soluble, which is advantageous for formulation and bioavailability. However, two compounds, C6 and C15, were categorized as poorly soluble, which could limit their practical application unless addressed by formulation strategies. Together, these findings suggest that the chemical profile of *A. cardamomum* seeds is largely consistent with drug-likeness criteria, although specific molecules may require optimization to overcome solubility or metabolic limitations.

**3.2.3.2 Lipinski’s rules of five**

Lipinski’s rule of five indicates that 20 of the 22 evaluated compounds meet the criteria for drug-likeness compounds ( Table 5). However, two compounds, C15 and C18, do not fully adhere to these criteria. C15 exhibits noncompliance due to the consensus log P value >5 (6.964573) and molar refractivity >130 (130.880981), which indicates potential challenges related to excessive lipophilicity and molecular size. LogP value indicates the level of lipophilicity, where high lipophilicity can result in quickly metabolized compounds, low solubility, and poorly absorbed ( Rutkowska et al., 2013). Molar refractivity values within the range of 40-130 suggest that a substance is likely to have good intestinal absorption and oral bioavailability ( Ya’u Ibrahim et al., 2020). Conversely, C18 fails to meet the criteria because of its H-bond receptor count >5 (6) and molar refractivity <40 (17.236599), indicating potential issues with polarity and molecular size. The H-bond acceptor and H-bond donor present in the structure of a therapeutic agent play a vital role in membrane transport, drug-protein interactions, distribution, and aqueous solubility ( Kenny, 2022). This evaluation provides valuable insights into the drug-like potential of the compounds from *A. cardamomum.*

**Table 5.** Lipinski’s rules of five results of *A.cardamomum* compounds.

Compound	Molecular weight (D) (g/mol)	Consensus log P	H-bond acceptor	H-bond donor	Molar refractory	Violation of Lipinski’s rules
**Standard**	**<500**	**≤5**	**<5**	**≤10**	**40-130**	**≤1**
C1	267	0.397590	4	2	64.743385	0
C2	168	-0.109530	3	3	42.125099	0
C3	124	1.178840	0	0	37.558395	0
C4	150	0.142820	2	2	39.077797	0
C5	179	0.843730	4	4	47.034191	0
C6	357	5.262740	0	0	105.343987	1
C7	226	1.603700	5	2	54.122093	1
C8	126	-0.663430	3	0	17.109299	0
C9	208	0.361710	2	2	54.749691	0
C10	134	1.803360	3	0	35.653496	0
C11	152	2.525599	1	0	50.743492	0
C12	138	2.837899	0	0	52.445992	0
C13	312	-0.053101	6	5	77.145782	1
C14	260	3.034269	5	1	72.196793	1
C15	339	6.964573	0	0	130.880981	2
C16	215	2.270190	3	1	63.245789	0
C17	324	3.838249	7	1	89.061790	1
C18	136	-0.715910	6	2	17.236599	2
C19	152	2.232710	1	1	50.070793	0
C20	361	3.902099	0	0	119.441383	0
C21	345	5.108690	2	0	112.781982	1
C22	113	0.992430	3	0	26.986998	1

**3.2.3.3 Toxicity prediction**

Based on the results of the toxicity analysis ( Table 6), three compounds, namely C6, C7, and C14, were found to be in the toxic level (class I-III). Overall, one compound (C14) is classified as class two toxicity (fatal by ingestion (5 < LD50 ≤ 50)), two compounds (C6 and C7) are classified as class III toxicity (toxic by ingestion (50 < LD50 ≤ 300)), twelve compounds (C1, C2, C4, C5, C8, C9, C11, C13, C15, C16, C20, and C21) are classified as class IV toxicity (harmful if swallowed (300 < LD50 ≤ 2000)), six compounds (C3, C10, C17, C18, C19, and C22) are classified as class V toxicity (may be harmful if swallowed (2000 < LD50 ≤ 5000)), and one compound (C12) are classified as class VI toxicity (non-toxic (LD50 > 5000)). Based on the analysis of organ toxicity, only 1 out of 22 compounds (C2) was reported to be active in the hepatotoxicity category. In the next category, 7 compounds (C4, C5, C6, C8, C10, C12, and C22) were reported as active carcinogenicity. Two out of 22 compounds (C4 and C8) were reportedly active in the mutagenicity. All evaluated compounds did not exhibit immunotoxicity or cytotoxicity. This is a positive sign that these compounds may not always risk genetic or cell damage, even if they are considered detrimental or possibly hazardous based on LD50 values [3]. Therefore, even if the chemicals from *A. cardamomum* have a variety of toxicity profiles, any prospective medicinal or pharmacological application must consider the unique risk factors associated with each component. Moreover, in vivo investigations are necessary to validate these in silico results and offer a more thorough comprehension of the safety and effectiveness of these substances.

**Table 6.** Toxicity level and organ target toxicity prediction of *A.cardamomum* compounds.

Compound	Predicted LD _50_	Predicted toxicity class	Average similarity	Organ toxicity				
				Hepatotoxicity	Carcinogenicity	Immunotoxicity	Mutagenicity	Cytotoxicity
C1	500 mg/kg	Four (harmful)	46.86%	Inactive	Inactive	Inactive	Inactive	Inactive
C2	350 mg/kg	Four (harmful)	100%	Active	Inactive	Inactive	Inactive	Inactive
C3	3500 mg/kg	Five (may be harmful)	68.6%	Inactive	Inactive	Inactive	Inactive	Inactive
C4	1300 mg/kg	Four (harmful)	46.57%	Inactive	Active	Inactive	Active	Inactive
C5	3900 mg/kg	Four (harmful)	82.22%	Inactive	Active	Inactive	Inactive	Inactive
C6	175 mg/kg	Three (toxic)	68.93%	Inactive	Active	Inactive	Inactive	Inactive
C7	237 mg/kg	Three (toxic)	100%	Inactive	Inactive	Inactive	Inactive	Inactive
C8	1484 mg/kg	Four (harmful)	44.01%	Inactive	Active	Inactive	Active	Inactive
C9	800 mg/kg	Four (harmful)	42.2%	Inactive	Inactive	Inactive	Inactive	Inactive
C10	4970 mg/kg	Five (may be harmful)	100%	Inactive	Active	Inactive	Inactive	Inactive
C11	470 mg/kg	Four (harmful)	100%	Inactive	Inactive	Inactive	Inactive	Inactive
C12	15380 mg/kg	Six (non-toxic)	100%	Inactive	Active	Inactive	Inactive	Inactive
C13	1520 mg/kg	Four (harmful)	100%	Inactive	Inactive	Inactive	Inactive	Inactive
C14	44 mg/kg	Two (fatal)	80.22%	Inactive	Inactive	Inactive	Inactive	Inactive
C15	1400 mg/kg	Four (harmful)	100%	Inactive	Inactive	Inactive	Inactive	Inactive
C16	1000 mg/kg	Four (harmful)	64.91%	Inactive	Inactive	Inactive	Inactive	Inactive
C17	3000 mg/kg	Five (may be harmful)	100%	Inactive	Inactive	Inactive	Inactive	Inactive
C18	2450 mg/kg	Five (may be harmful)	84.86%	Inactive	Inactive	Inactive	Inactive	Inactive
C19	2100 mg/kg	Five (may be harmful)	100%	Inactive	Inactive	Inactive	Inactive	Inactive
C20	500 mg/kg	Four (harmful)	51.4%	Inactive	Inactive	Inactive	Inactive	Inactive
C21	369 mg/kg	Four (harmful)	100%	Inactive	Inactive	Inactive	Inactive	Inactive
C22	4960 mg/kg	Five (may be harmful)	100%	Inactive	Active	Inactive	Inactive	Inactive

**3.2.4 Molecular docking**

The molecular docking analysis was conducted using the MOE application. To carry out a targeted molecular docking analysis, the grid box is accurately placed at the active site of each target protein. The grid box settings in the MOE application’s Site Finder feature were automatically modified according to the amino acid residues corresponding to the natural ligand of each protein. Molecular docking results include binding affinity (measured in kcal/mol), RMSD (Å), and visualization in two and three dimensions. A compound with an RMSD below 2.5 Å and a more favorable binding affinity (more negative) than the control ligand may indicate promising binding potential toward the target protein ( Table 7).

**Table 7.** Molecular docking results of SRC, TNF-α, Casp3, and EGFR protein.

Compound	SRC		TNF-α		Casp3		EGFR	
	BA	RMSD	BA	RMSD	BA	RMSD	BA	RMSD
Control	-7.89	1.56	-7.00	1.24	-6.53	1.64	-6.47	1.24
C1	-6.03	1.46	-5.83	2.41	-5.21	1.96	-5.05	1.62
C2	-5.86	0.96	-5.06	1.23	-4.72	1.32	-4.72	0.74
C3	-5.21	1.26	-4.77	1.53	-4.31	0.82	-5.04	0.96
C4	-5.71	0.99	-4.99	1.77	-4.70	0.85	-5.03	1.74
C5	-5.74	1.48	-5.14	1.15	-4.31	1.35	-4.48	1.26
C6	-6.70	1.13	6.32	1.31	-5.22	1.67	-6.02	1.61
C7	-6.37	1.40	-5.45	1.05	-4.46	1.27	-5.29	1.21
C8	-4.72	1.33	-4.26	1.83	-3.87	1.64	-4.51	1.24
C9	-6.38	1.11	-5.17	1.68	-4.76	0.97	-5.00	0.91
C10	-5.47	1.12	-4.70	1.33	-4.37	1.97	-4.90	1.30
C11	-6.13	1.34	-4.87	0.75	-4.18	1.68	-5.37	1.33
C12	-5.27	0.87	-4.45	1.74	-4.12	1.64	-5.00	1.89
C13	-7.27	1.78	-6.20	1.57	-5.90	1.90	-5.23	1.90
C14	-7.09	0.77	-5.67	1.50	-5.42	1.32	-5.47	1.66
C15	-7.83	1.91	-6.72	1.57	-6.02	1.85	-6.03	2.17
C16	-6.94	1.55	-5.65	1.64	-4.91	1.55	-5.26	1.41
C17	-7.84	1.82	-6.91	1.41	-6.70	1.51	-6.20	1.58
C18	-3.65	1.50	-4.31	0.83	-3.98	1.11	-4.19	1.15
C19	-5.55	1.73	-4.99	1.80	-4.55	1.47	-4.48	1.23
C20	-8.58	1.71	-6.88	2.00	-6.29	1.42	-6.43	0.90
C21	-7.70	2.20	-6.62	1.59	-6.34	1.91	-6.38	1.90
C22	-5.15	0.93	-4.47	1.09	-4.05	1.10	-4.52	0.76

**3.2.4.1 Molecular docking of SRC protein**

The molecular docking between SRC and *A. cardamomum* seed extract revealed that the compounds have RMSD values below 2Å. Based on the binding affinity, C20 exhibited the most favorable binding affinity (-8.58 kcal/mol), which was lower than that of the control ligand (-7.89 kcal/mol) ( Table 7). C20 formed three interactions: one acidic hydrophilic (Asp404) and two greasy hydrophobic interactions (Ala390 and Phe405). Control ligand formed three interactions: one acidic hydrophilic (Asp404), one basic hydrophilic (Arg388), and one greasy hydrophobic interaction (Val281). These results showed that C20 interacts with Asp404, the same amino acid residue with which the control ligand interacts ( Table 8). These findings suggest that C20 may interact favorably with the SRC active site and could be a candidate for further investigation.

**Table 8.** Molecular docking result of SRC protein.

Ligand	2D Interaction	3D Interaction
**Control**	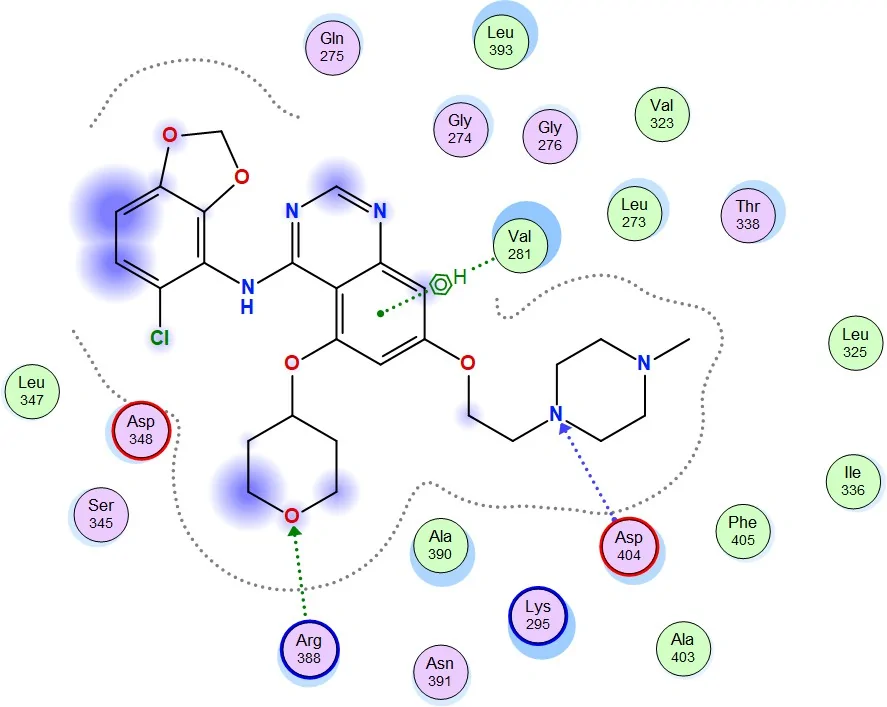	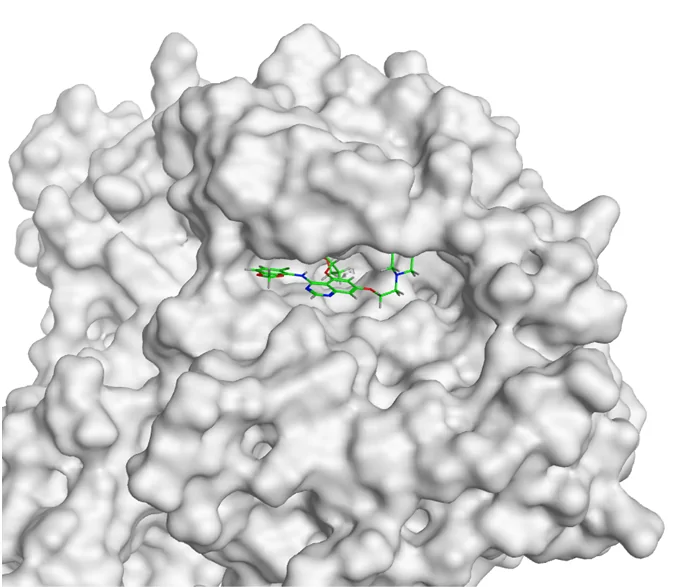
**C20**	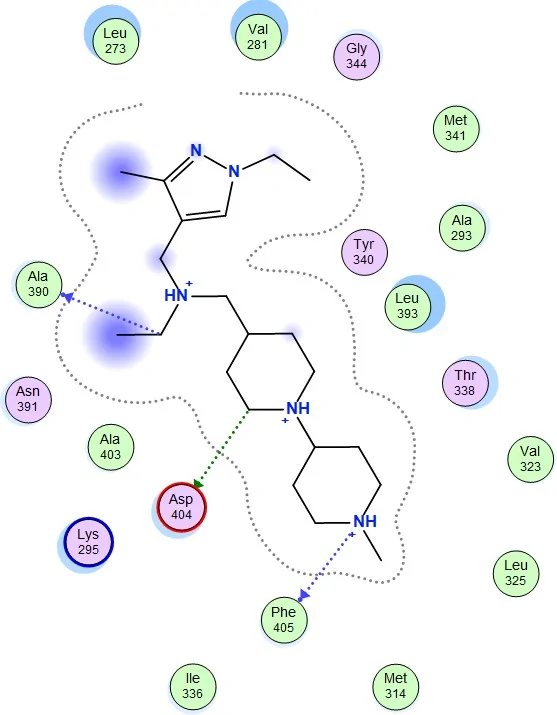	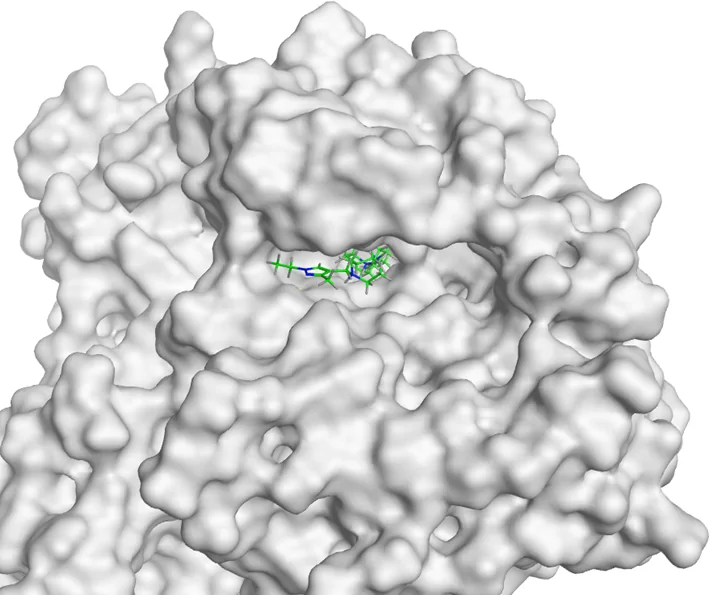

**3.2.4.2 Molecular docking of TNF-α protein**

From the molecular docking result, C17 attaches to the TNF-α active site with minimal deviation, marked by RMSD < 2Å (1.41 Å). C17 (-6.91 kcal/mol) has a binding affinity close to the control ligand’s (-7.00 kcal/mol) ( Table 7). C17 formed three polar hydrophilic interactions: TyrC119, TyrA119 and TyrA151. Meanwhile, the control ligand formed two polar hydrophilic interactions: TyrA119 and TyrA151. These showed that C17 and the control ligand interact with two similar amino acid residues of TNF-α (TyrA119 and TyrA151) ( Table 9). Therefore, C17 demonstrated favorable binding interactions with the TNF-α active site and may be a candidate for further investigation.

**Table 9.** Molecular docking result of TNF-α protein.

Ligand	2D Interaction	3D Interaction
**Control**	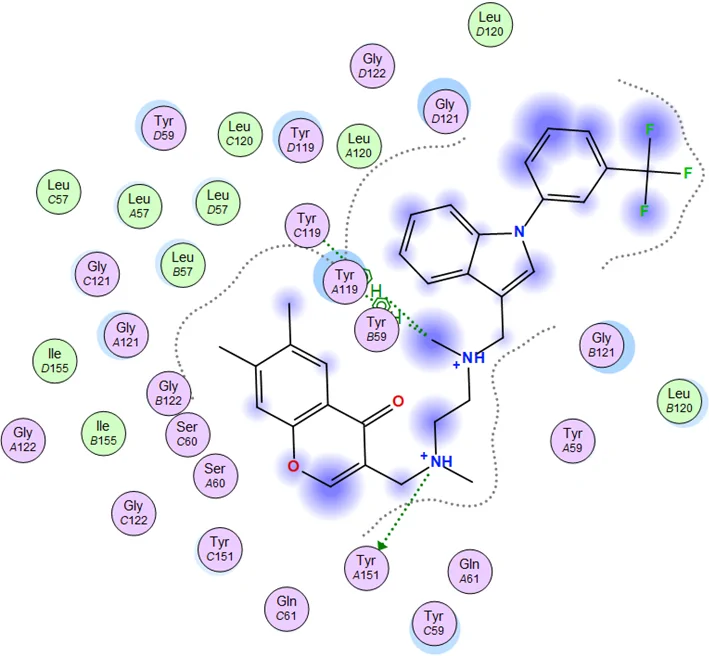	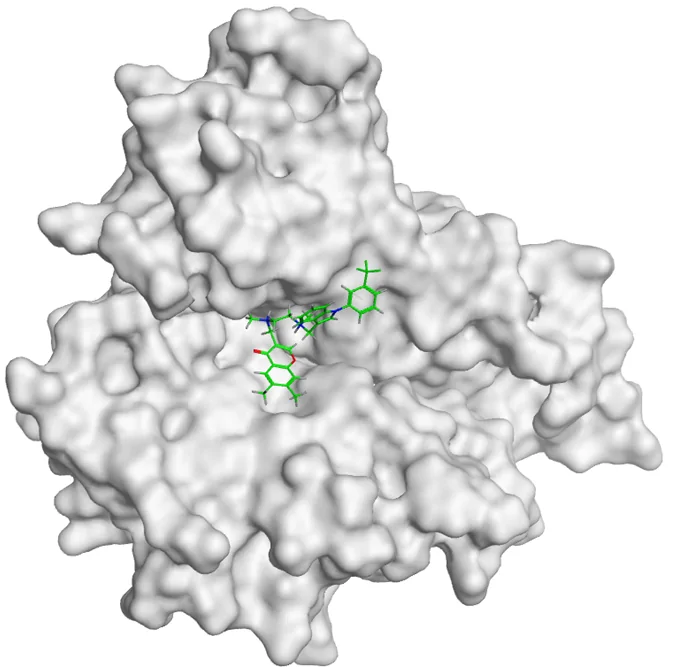
**C17**	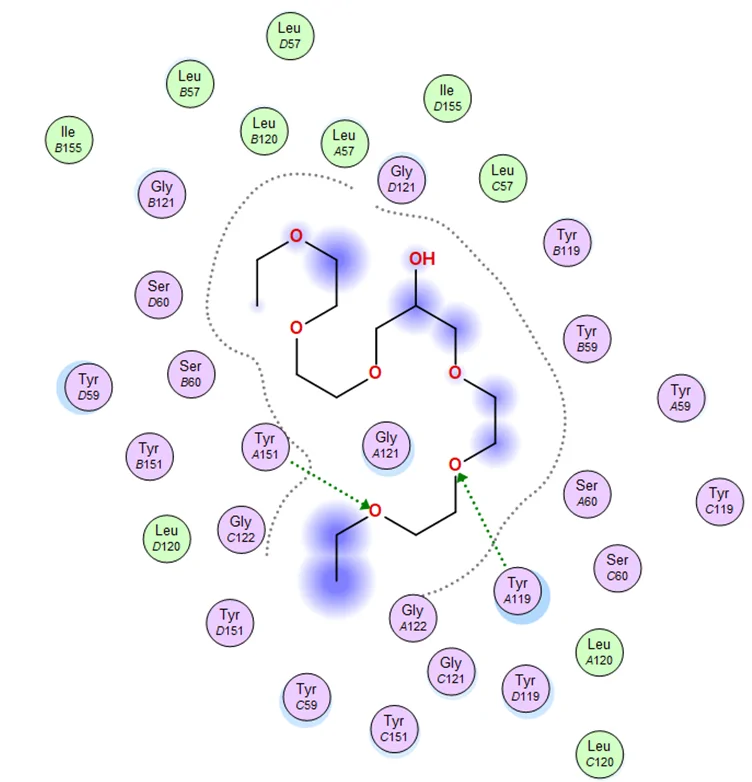	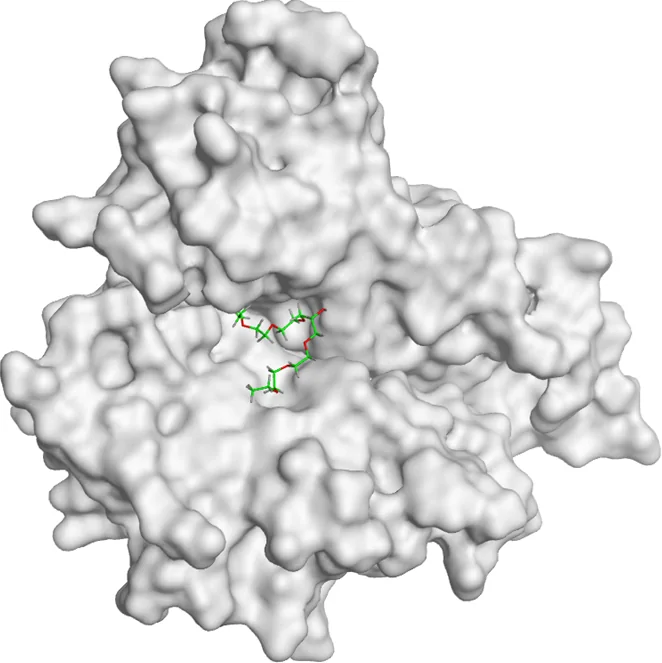

**3.2.4.3 Molecular docking of Casp3 protein**

Based on the binding affinity, C17 exhibited the most favorable binding affinity among the compounds of *A. cardamomum* and the control ligand (-6.70 kcal/mol) ( Table 7). The 2D interaction visualization revealed that C17 formed three interactions with the active site of casp3: one basic hydrophilic (Arg207), one polar hydrophilic (Asn208), and one greasy hydrophobic (Trp214) interaction. On the other hand, casp3 inhibitors formed one basic hydrophilic (Arg207) and two polar hydrophilic interactions (Thr62, Ser209) with the active site of casp3. This result showed that C17 and casp3 inhibitors formed one similar interaction with the active site of casp3 (Arg207) ( Table 10). These interactions suggest that C17 may exhibit favorable binding toward the casp3 active site and warrant further investigation.

**Table 10.** Molecular docking results of casp3 protein.

Ligand	2D Interaction	3D Interaction
**Control Drug**	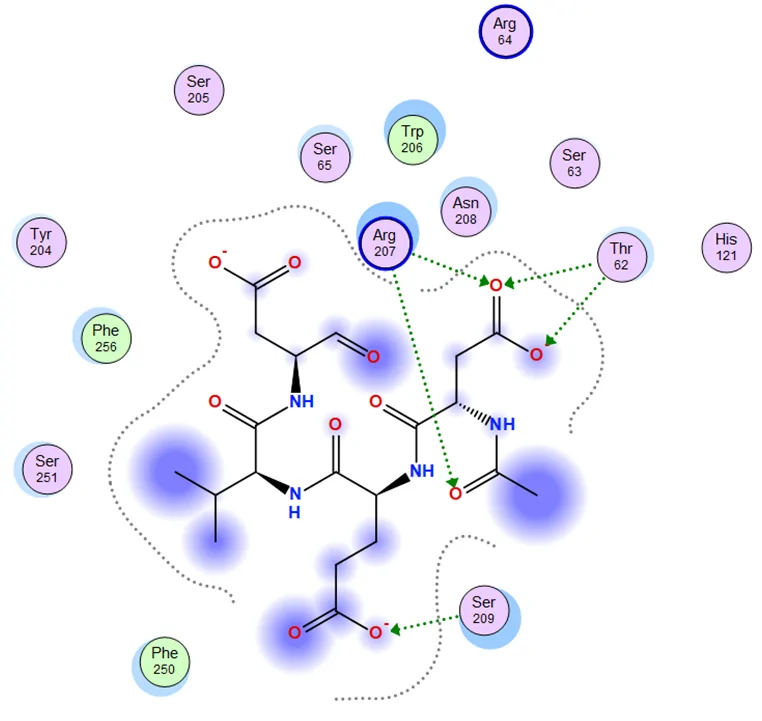	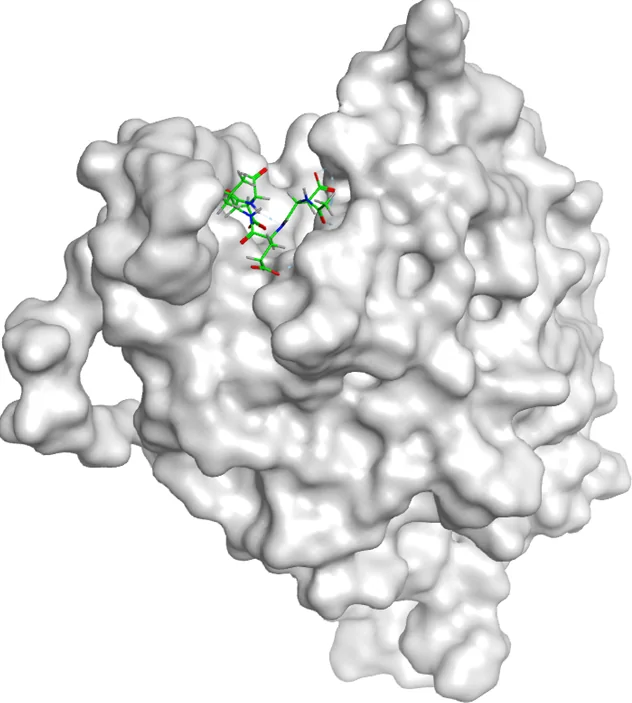
**C17**	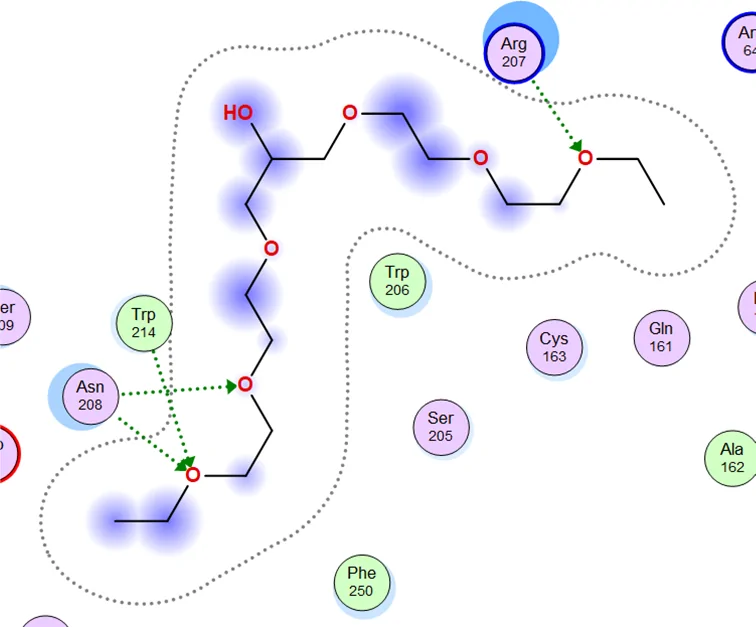	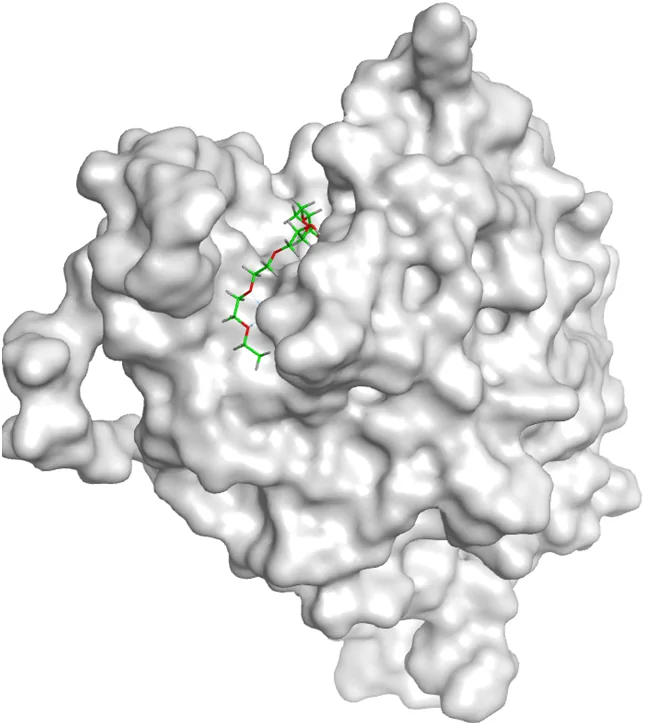

**3.2.4.4 Molecular docking of EGFR protein**

All compounds of *Amomum cardamomum* have RMSD values below 2 Å, except C15. Regarding binding affinity, C20 (-6.43 kcal/mol) has a binding affinity close to the control ligand (-6.47 kcal/mol) as the control ligand ( Table 7). Although the binding affinity of C20 was slightly lower than that of the control ligand, the values remained comparable. The 2D interaction visualization showed that C20 formed two acidic hydrophilic interactions with the active site of EGFR (Asp831 and Glu738). Although the binding affinity of C20 was slightly lower than that of the control ligand, the values remained comparable. The 2D interaction visualization showed that C20 formed two acidic hydrophilic interactions with the active site of EGFR (Asp831 and Glu738). Meanwhile, the control ligand formed one basic hydrophilic interaction (Lys721) and one greasy hydrophobic interaction (Phe699). Although C20 does not have identical amino acid residues as the control ligand, the 3D interaction visualization suggested that C20 may occupy a binding region similar to that of the control ligand on the EGFR active site ( Table 11).

**Table 11.** Molecular docking result of EGFR protein.

Ligand	2D Interaction	3D Interaction
**Control**	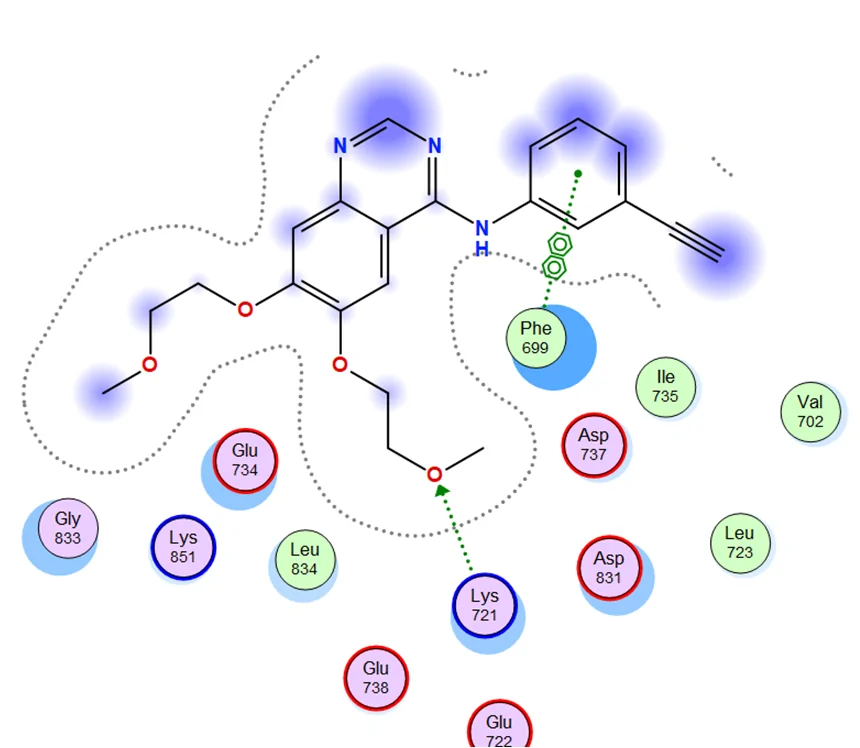	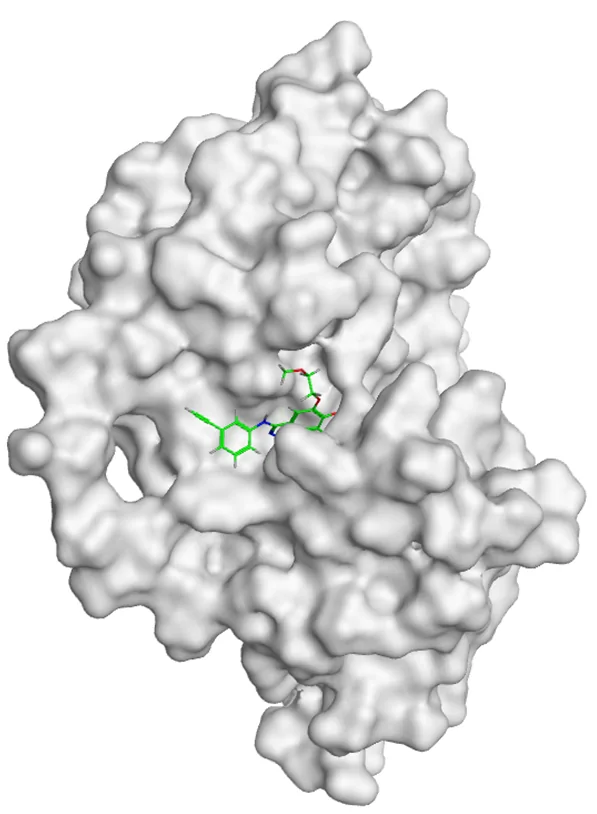
**C20**	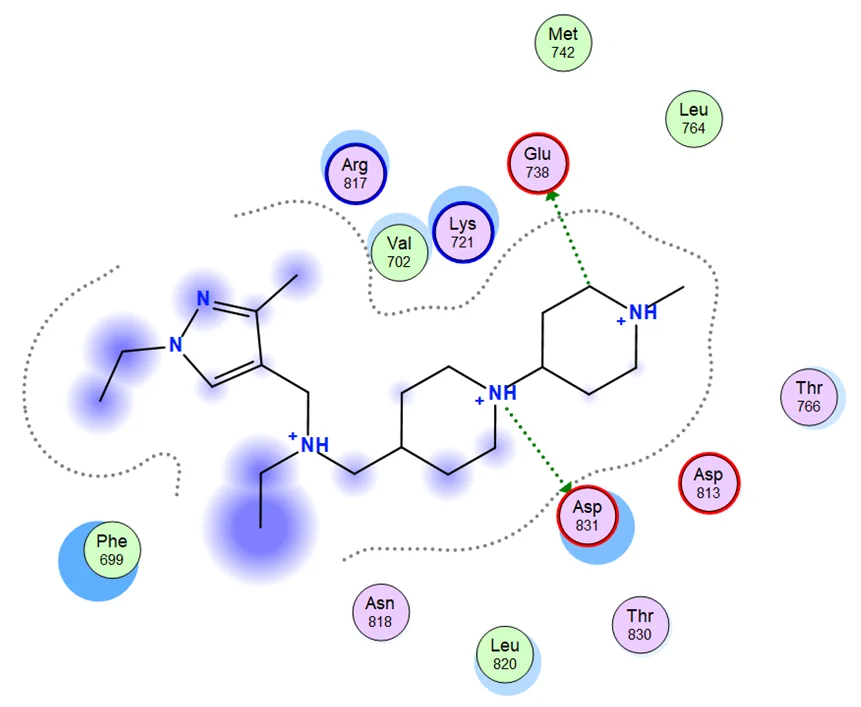	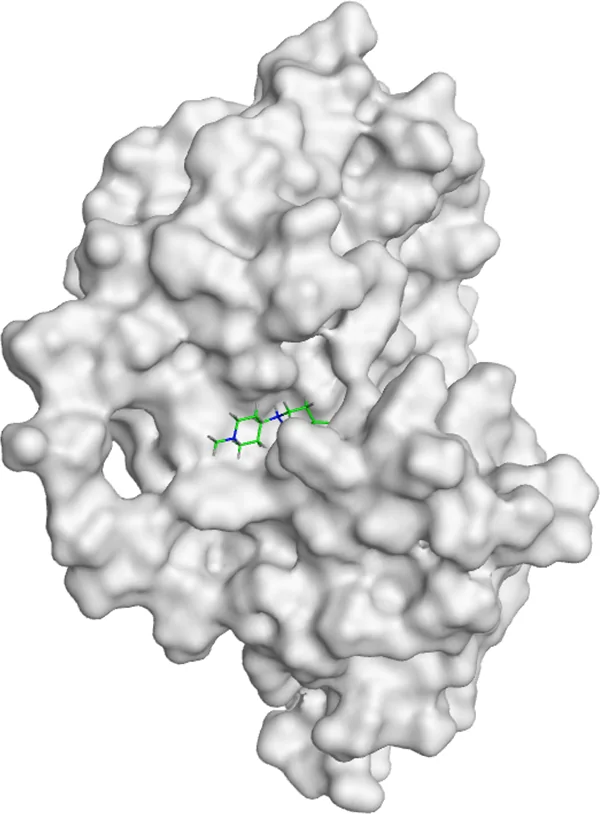

**3.2.5 Molecular dynamic simulation**

Molecular dynamics simulations were performed to evaluate the structural stability of the protein–ligand complexes over a 50 ns time scale. The analysis focused on root mean square deviation (RMSD), radius of gyration (Rg), and solvent-accessible surface area (SASA) to assess the overall conformational stability and compactness of each system ( Figure 4).

**Figure 4.**
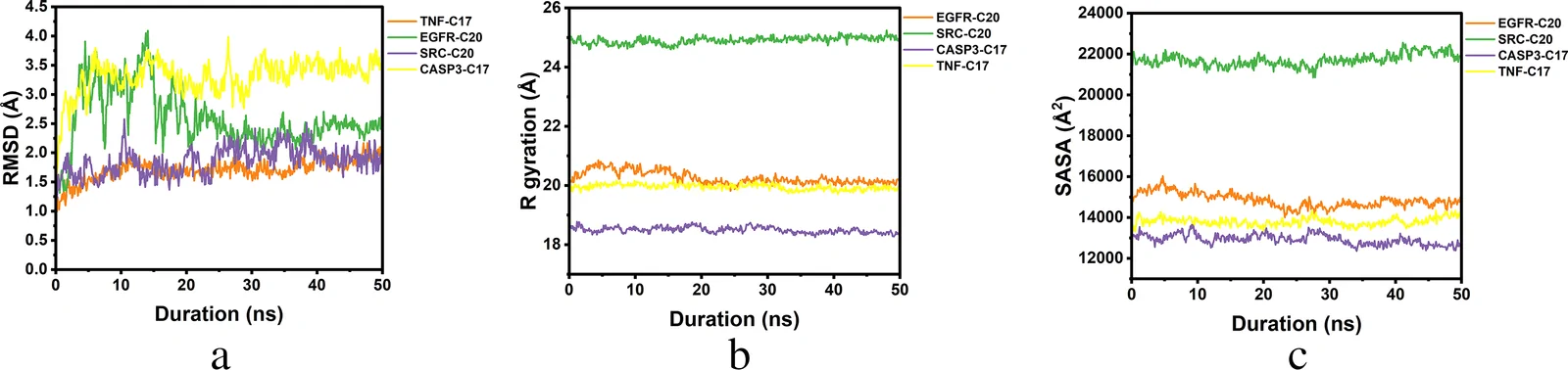
Molecular Dynamic. (a) RMSD; (b) R gyration; and (c) SASA.

The RMSD analysis ( Figure 4a) indicates that TNFα–C17 exhibits stable behavior, with values consistently around 1.5–2.0 Å throughout the 50 ns simulation. SRC–C20 shows similar stability with RMSD values fluctuating around 1.5–2.0 Å, with slightly higher variation compared to TNFα–C17. EGFR–C20 demonstrates higher RMSD values (2.5–4.0 Å), with notable fluctuations during the first 25 ns before reaching a more stable plateau around 2.5 Å. CASP3–C17 shows relatively higher RMSD values ranging between 3.0–3.5 Å throughout the simulation period. Overall, all complexes reach a relatively stable state after approximately 25 ns, although each system exhibits different stability ranges.

Furthermore, the radius of gyration (Rg) analysis ( Figure 4b) shows that all protein–ligand complexes maintain relatively consistent compactness throughout the simulation. CASP3–C17 displays the lowest Rg values, suggesting a more compact structure, while TNFα–C17 and EGFR–C20 show slightly higher but stable Rg profiles. SRC–C20 exhibits comparatively higher Rg values, indicating a relatively larger but stable structural conformation over time.

Moreover, the solvent-accessible surface area (SASA) results further indicate that all complexes maintain stable solvent exposure during the simulation period. SRC–C20 shows the highest SASA values, while CASP3–C17 exhibits the lowest SASA values among the four systems. TNFα–C17 and EGFR–C20 display intermediate SASA values, with no major fluctuations observed. Overall, RMSD, Rg, and SASA results collectively suggest that all complexes maintain structural stability under simulation conditions without large conformational disruptions.

## 4. Discussion

This study demonstrated the therapeutic potential of *A. cardamomum* through a combination of LC-MS analysis, pharmacokinetic and pharmacodynamic prediction, network pharmacology, molecular docking, and molecular dynamic simulation. LC-MS analysis identified 22 unique compounds in the seed extract, showcasing a chemically diverse profile with various molecular weights and retention times. The protein-protein interaction (PPI) network studies revealed key protein targets for *A. cardamomum* compounds, including SRC, EGFR, TNF-α, and Casp3, all of which play critical roles in breast cancer progression.

SRC, a non-receptor tyrosine kinase and the first proto-oncogene identified in mammalian cells plays a crucial role in breast cancer (BC) by promoting tumor initiation, growth, metastasis, and drug resistance ( Luo et al., 2022). SRC is overexpressed or activated in various stages of BC, where it enhances signaling pathways that promote cell proliferation, survival, and motility, contributing to tumorigenesis. SRC activation leads to the phosphorylation of epidermal growth factor receptor (EGFR) ( Finn, 2008; Pelaz & Tabernero, 2022; Raji et al., 2024; Wheeler et al., 2009). Additionally, SRC interacts with HER2, amplifying signaling through pathways like FAK and STAT3, which increase the metastatic potential and contribute to resistance against HER2-targeted therapies ( Raji et al., 2024). Inhibition of SRC has been shown to disrupt these pathways and reduce tumorigenesis, making SRC a critical target for therapeutic intervention in BC. One compound that can inhibit SRC activity is Quinazoline. Quinazoline exerts its effects by binding to the ATP-binding pocket of SRC, leading to the inhibition of its kinase activity. This interaction disrupts signaling pathways essential for tumor cell survival and growth, such as the PI3K-AKT and MAPK pathways ( Emami et al., 2022).

Tumor necrosis factor (TNF-α) is a pro-inflammatory cytokine that plays a significant role in developing and progressing breast cancer. TNF-α is involved in carcinogenesis by activating several signaling pathways, including the TNF receptor 1 (TNFR1) pathway, which leads to the upregulation of oncoproteins such as HBXIP. This activation promotes cell proliferation, survival, and migration, contributing to tumorigenesis. Elevated TNF-α levels are often observed in breast cancer tissues, and its signaling is linked to increased metastasis and drug resistance, making TNF-α a critical factor in BC progression. Inhibition of TNF-α has been shown to reduce cancer cell proliferation, suppress tumor growth, and decrease metastatic potential. Consequently, TNF-α inhibitors are being explored as potential therapeutic agents for BC treatment. Targeting TNF-α makes it possible to disrupt its downstream signaling pathways, including the NF-κB and MAPK pathways, crucial for maintaining tumor cell survival and inducing inflammatory responses that support tumor growth ( Cai et al., 2017; Mercogliano et al., 2020). Given the importance of TNF-α in breast cancer progression, targeting its activity offers a promising strategy to mitigate carcinogenesis and enhance the efficacy of existing therapies.

Caspase-3 (Casp3) plays a complex role in breast cancer, acting as a key apoptosis regulator, but its dysregulation can contribute to tumor progression and chemotherapy resistance ( Pu et al., 2017). High Casp3 expression is associated with poor prognosis, particularly in HER2-positive tumors and specific patient populations, while Casp3 deficiency reduces tumorigenesis in preclinical models ( Pu et al., 2017; Zhu et al., 2024). Casp3 activation is crucial for apoptosis, but its activity can be inhibited by factors such as the X-linked inhibitor of apoptosis protein (XIAP), which prevents its activation and subsequent cell death ( Yosefzon et al., 2018). These findings highlight Casp3’s dual role in promoting tumorigenesis and resistance to treatment, suggesting it could be a target for therapeutic strategies in breast cancer.

Epidermal growth factor receptor (EGFR) is a key player in breast cancer progression, as it regulates critical processes like cell proliferation, survival, migration, and differentiation. EGFR overexpression in breast cancer leads to the dysregulation of signaling pathways such as PI3K-AKT and MAPK, promoting tumorigenesis, metastasis, and resistance to apoptosis ( Sigismund et al., 2017). This is particularly evident in triple-negative breast cancer (TNBC), where EGFR overexpression is linked to poor prognosis. EGFR also interacts with other receptors, like HER2, amplifying tumor growth signals ( Masuda et al., 2012). Due to its central role, EGFR is potentially to be a target for therapeutic strategies.

Overall, the integrated in silico analysis suggests that compounds identified in the ethanolic extract of *A. cardamomum*, particularly C17 and C20, may interact with several breast cancer-related proteins involved in inflammation, apoptosis, and oncogenic signaling pathways. Molecular docking analysis demonstrated favorable binding interactions with key target proteins, while ADMET prediction indicated generally acceptable pharmacokinetic properties for several compounds. In addition, molecular dynamics simulations suggested relatively stable protein–ligand interactions under simulation conditions. Collectively, these findings provide preliminary computational evidence supporting the potential of *A. cardamomum* as a source of bioactive compounds for further breast cancer-related research.

## 5. Conclusion

This study demonstrated that compounds identified from the ethanolic extract of *A. cardamomum* seed may have potential anti-breast-cancer activity based on integrated in silico analyses. Network pharmacology analysis highlighted several important breast cancer-related proteins, including TNF, SRC, EGFR, and CASP3, which were associated with inflammation, apoptosis, and cancer-related signaling pathways. Among the identified compounds, C17 and C20 showed favorable interaction profiles and relatively stable protein–ligand complexes during molecular dynamics simulations. In addition, most compounds exhibited generally acceptable predicted pharmacokinetic and drug-likeness properties. As an in-silico approach, these findings primarily provide a preliminary prediction of the possible biological activity, interaction stability, and drug-likeness properties of the identified compounds. Therefore, further in vitro and in vivo studies are required to experimentally validate the predicted anticancer activity and assess their therapeutic potential in preclinical models.

## Ethics approval

Ethics and consent were not required.

## Data Availability

Zenodo: Exploration of *Amomum cardamomum* Seed-Derived Compounds: LC-MS Profiling, Network Pharmacology, and Computational Analyses for Innovative Multi-Targeted Breast Cancer Therapy. doi: https://doi.org/10.5281/zenodo.20282808 ( Cuandra, 2025) Ver2. This project contains the following underlying data:
• TNFA-KAPULAGA.xlsx • CASP3-KAPULAGA.xlsx • EGFR-KAPULAGA.xlsx • SRC-KAPULAGA.xlsx • Interpretation of LCMS-MS Kapulaga.pdf • Overall Centrality.csv Data are available under the terms of the Creative Commons Attribution 4.0 International license (CC-BY 4.0).
