# Supplementary material for: Exploration of Compounds Identified from Amomum cardamomum Seed Extract: LC-MS Profiling, Network Pharmacology, and Computational Analyses for Innovative Multi-Targeted Breast Cancer Therapy
Source: F1000Res. 2026 Aug 6;14:1374. Originally published 2025 Dec 8. [Version 2] doi: 10.12688/f1000research.168214.2 (PMC13684023; doi:10.12688/f1000research.168214.2)
Supplement: Peer review history

# Reviewer report: Reviewer response for version 2

**DOI:** 10.5256/f1000research.201810.r526865

**Published:** 2026-10-20

**Authors:** Massar Shekhan

**Affiliations:** 1 The American University of Iraq, Baghdad, Iraq

Major Concerns

**1. Lack of Breast Cancer Subtype Specificity and Investigation of Drug Resistance**

 The authors emphasize therapeutic resistance as a major challenge in breast cancer treatment. However, the study does not identify which molecular subtype of breast cancer is being targeted (ER-positive, HER2-positive, or triple-negative breast cancer), nor does it investigate a specific mechanism of drug resistance. Given the molecular heterogeneity of breast cancer, the biological relevance of the selected targets (SRC, EGFR, TNF-α, and CASP3) may differ significantly among breast cancer subtypes.

 The authors should clarify the biological rationale for selecting these targets and explain how their findings relate to specific breast cancer subtypes and mechanisms of therapeutic resistance. If the study does not directly investigate drug resistance, the authors must acknowledge this limitation and avoid suggesting that the identified compounds could overcome therapeutic resistance without supporting evidence.

**2. Overinterpretation of Molecular Docking Results and Lack of Functional Validation**

 The authors rely heavily on molecular docking and molecular dynamics simulations to propose C17 and C20 as potential anticancer compounds. However, molecular docking predicts possible protein–ligand interactions and does not demonstrate target inhibition, biological activity, or therapeutic efficacy. Similarly, molecular dynamics simulations provide information about structural stability but cannot establish anticancer activity.

 Of particular concern is the interpretation of CASP3 targeting. Since caspase-3 is a key executioner of apoptosis, the authors must clarify whether the proposed interaction would potentially activate or inhibit its activity. Binding alone cannot establish a beneficial anticancer mechanism.

 The authors should revise their conclusions to distinguish computational predictions from experimentally demonstrated biological effects. Functional validation through enzyme activity assays, apoptosis assays, and breast cancer cell models is strongly recommended. If experimental validation is beyond the scope of this study, the authors must explicitly acknowledge this limitation and restrict their conclusions to identifying potential compounds for future investigation.

Is the work clearly and accurately presented and does it cite the current literature?

Yes

If applicable, is the statistical analysis and its interpretation appropriate?

Partly

Are all the source data underlying the results available to ensure full reproducibility?

Partly

Is the study design appropriate and is the work technically sound?

Partly

Are the conclusions drawn adequately supported by the results?

No

Are sufficient details of methods and analysis provided to allow replication by others?

Partly

Reviewer Expertise:

15

I confirm that I have read this submission and believe that I have an appropriate level of expertise to confirm that it is of an acceptable scientific standard, however I have significant reservations, as outlined above.

# Reviewer report: Reviewer response for version 2

**DOI:** 10.5256/f1000research.201810.r508610

**Published:** 2026-08-25

**Authors:** Hayarpi Javrushyan

**Affiliations:** 1 Research Institute of Biology, Yerevan State University, Yerevan, Armenia

I have reviewed the revised version and the authors' responses to my previous comments.

 I am satisfied that my main concerns have been adequately addressed, particularly regarding the compound-labeling inconsistencies, network pharmacology analysis, docking validation, MD analysis, ADMET interpretation, and the overall interpretation of the in-silico findings.

 I have only one minor recommendation: since the authors clarify that the LC–MS compounds represent putative annotations based on database matching, this terminology and the corresponding limitation should be stated consistently throughout the manuscript, particularly as these annotations form the basis for the subsequent computational analyses.

 Subject to this minor point, I have no further major concerns and am happy to change my previous status to ***Approved.***

Is the work clearly and accurately presented and does it cite the current literature?

Partly

If applicable, is the statistical analysis and its interpretation appropriate?

Not applicable

Are all the source data underlying the results available to ensure full reproducibility?

Partly

Is the study design appropriate and is the work technically sound?

Partly

Are the conclusions drawn adequately supported by the results?

Partly

Are sufficient details of methods and analysis provided to allow replication by others?

Partly

Reviewer Expertise:

NA

I confirm that I have read this submission and believe that I have an appropriate level of expertise to confirm that it is of an acceptable scientific standard.

# Reviewer report: Reviewer response for version 1

**DOI:** 10.5256/f1000research.185380.r473521

**Published:** 2026-04-20

**Authors:** Hayarpi Javrushyan

**Affiliations:** 1 Research Institute of Biology, Yerevan State University, Yerevan, Armenia

This manuscript presents an integrated in silico investigation of *Amomum cardamomum* seed-derived compounds as potential multi-target agents against breast cancer, combining LC–MS profiling, network pharmacology, ADMET prediction, molecular docking, and molecular dynamics simulations. The topic is relevant and timely, and the overall approach—linking phytochemical profiling with computational analyses—is appropriate and of potential interest.

 However, in my view, the manuscript in its current form has several important methodological limitations and internal inconsistencies that significantly weaken the strength of the conclusions.

 One of the main concerns relates to the inconsistency between the compounds highlighted as leads and those subjected to molecular dynamics simulations. While C17 and C20 are presented throughout as the most promising candidates, the MD simulations were performed on different compounds (C18 and C21). The abstract and conclusions nevertheless state that the stability of C17 and C20 was confirmed by MD simulations, which is not supported by the data shown. This is not a minor issue and needs to be resolved either by performing simulations on the correct compounds or by revising the manuscript to ensure internal consistency.

 More generally, the manuscript tends to overinterpret in silico results. Statements describing compounds as “specific inhibitors” or “therapeutic candidates” go beyond what can be concluded from docking and MD simulations alone. These approaches provide useful predictions of potential interactions, but they do not demonstrate biological activity or efficacy. The conclusions should therefore be tempered, and the predictive nature of the findings should be clearly acknowledged.

 Another point that requires attention is the identification of compounds by LC–MS. At present, the manuscript does not provide sufficient validation (e.g., MS/MS fragmentation data, reference standards, or clear database matching criteria). In addition, some of the reported compounds appear unusual for plant-derived metabolites, which raises the possibility of misannotation or contamination. Since all downstream analyses depend on the correctness of compound identification, this aspect needs to be clarified.

 The network pharmacology analysis would also benefit from more stringent filtering. The use of very large gene sets from databases such as GeneCards and CTD makes the overlap relatively non-specific and difficult to interpret biologically. Applying stricter criteria and including pathway enrichment analysis would strengthen this part of the study.

 From a methodological perspective, the docking procedure lacks validation details (e.g., redocking), and there is some inconsistency in the reported RMSD thresholds. Similarly, the molecular dynamics analysis relies mainly on RMSD, without additional parameters such as RMSF or interaction analysis, which limits the strength of the conclusions regarding complex stability.

 There are also a few inconsistencies in data interpretation, for example, in the discussion of ADMET results (including BBB permeability) and in the description of transporter-related properties. These should be carefully revised to ensure that all statements are directly supported by the presented data. Minor issues include typographical errors, inconsistent nomenclature, and figures that could be improved for clarity.

 Thus, while the study has potential and follows a commonly used computational workflow, the current version requires substantial revision to ensure methodological transparency, internal consistency, and appropriately supported conclusions.

Is the work clearly and accurately presented and does it cite the current literature?

Partly

If applicable, is the statistical analysis and its interpretation appropriate?

Not applicable

Are all the source data underlying the results available to ensure full reproducibility?

Partly

Is the study design appropriate and is the work technically sound?

Partly

Are the conclusions drawn adequately supported by the results?

Partly

Are sufficient details of methods and analysis provided to allow replication by others?

Partly

Reviewer Expertise:

Biochemistry, Cancer Biology

I confirm that I have read this submission and believe that I have an appropriate level of expertise to confirm that it is of an acceptable scientific standard, however I have significant reservations, as outlined above.

# Response

**Published:** 2026-05-21

**Authors:** Dessy Arisanty

**Affiliations:** Universitas Andalas, Padang, West Sumatra, Indonesia

We sincerely thank the reviewer for the thorough and constructive evaluation of our manuscript. We have carefully revised the manuscript to address all raised concerns. Point-by-point responses are provided below.

 1. Internal inconsistency between highlighted compounds and MD simulations

**Response: **We acknowledge the inconsistency in compound labeling between the docking and MD simulation sections. This issue was caused by a labeling error during figure and software annotation preparation. The molecular dynamics simulations were performed on the intended lead compounds (C17 and C20), but incorrect labels were mistakenly written (C18 and C21). These labeling errors in figure and result have now been corrected.

 2. Overinterpretation of in silico findings

**Response: **The manuscript has been revised to tone down the language and have been replaced with more appropriate wording. The conclusions now clearly emphasize the predictive and exploratory nature of the in silico analyses.

 3. LC–MS compound identification concerns

**Response: **Compound identification was performed based on accurate mass (m/z), retention time, and molecular formula prediction using LC–MS data. The identification of compounds was carried out through database matching; therefore, the results represent putative annotations.

 4. Network pharmacology analysis concerns

**Response: **We have refined the target filtering strategy to improve specificity of the overlapping genes obtained from GeneCards and CTD, ensuring more focused disease-related targets. We also strengthened the biological interpretation by performing GO and KEGG pathway enrichment analyses to better characterize the functional roles of the shared targets. These analyses helped to identify key biological processes and signaling pathways related to inflammation, apoptosis, kinase regulation, extracellular matrix remodeling, and cancer progression.

 5. Docking and MD methodology limitations

**Response: **In the revised manuscript, we added a description of the docking validation procedure in the Methods section, which was not sufficiently explained in the previous version. Specifically, we clarified that native ligands were isolated using BIOVIA Discovery Studio Visualizer version 2024 and re-docked for validation, with all RMSD values obtained below 2.0 Å, indicating acceptable docking reliability. The molecular dynamics (MD) analysis section was also revised to improve clarity and consistency. In addition to RMSD analysis, radius of gyration (Rg) and solvent-accessible surface area (SASA) analyses were included to provide a more comprehensive evaluation of complex stability during the simulation.

 6. Data interpretation inconsistencies

**Response: **We carefully reviewed and corrected inconsistencies in the ADMET section, including BBB permeability and transporter-related predictions (e.g., OCT2 substrate vs inhibitor). All statements have been revised to match the values presented in Table 3.

 7. Minor issues

**Response: **We have corrected typographical errors and improved figure clarity and labeling to enhance readability.

# Reviewer report: Reviewer response for version 1

**DOI:** 10.5256/f1000research.185380.r445457

**Published:** 2026-01-28

**Authors:** Jun Sang Yu

**Affiliations:** 1 Jeju National University, Jeju-si, Jeju-do, South Korea

I have reviewed the above manuscript. The study combines LC–MS-based compound identification with network pharmacology, molecular docking, and molecular dynamics simulations to explore potential bioactive compounds. While the overall in silico workflow is familiar and generally acceptable for a descriptive study, several issues related to data consistency, methodological correctness, and result interpretation require clarification and correction. These concerns are largely addressable without additional experiments; therefore, I recommend **minor revision**.

**1) Table 2: duplicated compound index and incorrect ion notation**

 In Table 2, compound **C22 appears twice**, indicating an indexing or annotation error that should be corrected. In addition, the reported ion form **[M–H]+ is not standard**. In mass spectrometry, positive ion mode is conventionally expressed as **[M+H]+**, whereas **[M–H]−** corresponds to negative ion mode. Since the manuscript states that the analysis was performed in positive ion mode, the ion notation should be revised for correctness and clarity.

**2) Figure 3 lacks clear scientific meaning and interpretability**

 It is difficult to understand what Figure 3 is intended to demonstrate. The presented network visualization does not convey quantitative or interpretable information, and no network metrics or analytical conclusions are directly supported by the figure. As currently shown, the figure appears largely illustrative rather than informative. The authors should clarify its purpose, improve its interpretability, or reconsider its inclusion.

**3) Questionable classification of several compounds as “seed-derived”**

 The manuscript repeatedly refers to the identified compounds as *seed-derived*. However, several compounds listed in Table 2 are difficult to reconcile with known plant secondary metabolites and may reflect contamination or database misannotation rather than genuine seed constituents. Without additional justification or validation, the use of the term *seed-derived compounds* appears overstated and should be reconsidered or more carefully qualified.

**4) Misinterpretation of ADMET results: OCT2 substrate vs inhibitor**

 In the ADMET analysis, Table 3 reports OCT2 **substrate** status, yet the manuscript text describes this as **inhibitory activity** toward OCT2. These two terms have fundamentally different pharmacokinetic implications. The text should be corrected to accurately reflect the reported data.

**5) Inconsistency between BBB permeability results and conclusions**

 The conclusions state that both **C17 and C20 are BBB-permeant**, whereas Table 3 reports **C17 as BBB permeant = No**. This represents a direct inconsistency between the tabulated results and the written conclusions. The authors should revise the conclusion or clarify the basis for this claim.

**6) Mismatch between highlighted lead compounds and MD simulations**

 The manuscript emphasizes **C17 and C20** as key compounds; however, the molecular dynamics simulations were performed on **C18 and C21**. Furthermore, the abstract states that MD simulations confirmed the stability of C17 and C20, which is not supported by the presented MD results. This discrepancy between the abstract, methods, and results should be resolved to ensure internal consistency.

 Overall, the manuscript presents a conventional in silico screening workflow, but several internal inconsistencies, incorrect interpretations, and unclear presentations undermine the clarity and reliability of the conclusions. These issues appear correctable through careful revision of tables, figures, and text, and therefore a decision of *minor revision* is appropriate.

Is the work clearly and accurately presented and does it cite the current literature?

Yes

If applicable, is the statistical analysis and its interpretation appropriate?

Partly

Are all the source data underlying the results available to ensure full reproducibility?

Partly

Is the study design appropriate and is the work technically sound?

Yes

Are the conclusions drawn adequately supported by the results?

Partly

Are sufficient details of methods and analysis provided to allow replication by others?

Yes

Reviewer Expertise:

Pharmacokinetics, ADME, Pharmaceutical analysis, Mass spectrometry, GNPS

I confirm that I have read this submission and believe that I have an appropriate level of expertise to confirm that it is of an acceptable scientific standard, however I have significant reservations, as outlined above.

# Response

**Published:** 2026-05-21

**Authors:** Dessy Arisanty

**Affiliations:** Universitas Andalas, Padang, West Sumatra, Indonesia

We sincerely thank the reviewer for the careful evaluation and constructive comments. We have revised the manuscript accordingly and addressed all concerns raised. Detailed responses are provided below.

1. **Response: **The duplicated “C22” in Table 2 was caused by a typographical error in the compound codename numbering. The first “C22” should have been written as “C21.” These labels were only internal codenames used to simplify discussion in the manuscript and did not affect the compound identification or subsequent analyses. The numbering has now been corrected throughout the revised manuscript. In addition, the ion notation has also been corrected. Since the LC–MS analysis was performed in positive ion mode, the notation was revised from **“[M–H]+”** to the correct form **“[M+H]+.**”
2. **Response: **The PPI analysis section and figure presentation have been revised to improve clarity and interpretability. In the revised manuscript, we added a clearer explanation of the workflow used to obtain the overlapping targets and separated the visualization into different panels, including the Venn diagram, PPI network, and topological analysis. We also added quantitative network analysis using CytoNCA and PCA-based centrality scoring to better support the identification of hub proteins. We also add GO and KEGG enrichment for further analysis. The revised results now explain that the overlapping targets are involved in interconnected pathways related to inflammation, apoptosis, kinase regulation, and extracellular matrix remodeling.
3. **Response: **The terminology throughout the manuscript has been revised to avoid overstating the origin of the identified compounds. Since the compounds were putatively identified from the ethanolic extract of *A. cardamomum *using LC–MS profiling, terms such as “seed-derived compounds” were replaced with more appropriate wording, such as “compounds identified in the extract", including in the article title.
4. **Response: **The text describing the ADMET results has been corrected to match the information presented in Table 3. The previous version incorrectly described OCT2 substrate prediction as inhibitory activity. This has now been revised.
5. **Response: **The statement regarding BBB permeability has been corrected to match the results presented in Table 3. The conclusion section has been revised.
6. **Response: **This issue was caused by a labeling error during figure and software annotation preparation. The molecular dynamics simulations were performed on the intended lead compounds (C17 and C20), but incorrect labels were mistakenly written. These labeling errors in figure and result have now been corrected.
